# Bacterial Community Composition in the Sea Surface Microlayer Off the Peruvian Coast

**DOI:** 10.3389/fmicb.2018.02699

**Published:** 2018-11-15

**Authors:** Birthe Zäncker, Michael Cunliffe, Anja Engel

**Affiliations:** ^1^GEOMAR – Helmholtz Centre for Ocean Research Kiel, Kiel, Germany; ^2^Marine Biological Association of the United Kingdom, Plymouth, United Kingdom; ^3^Marine Biology and Ecology Research Centre, School of Biological and Marine Sciences, Plymouth University, Plymouth, United Kingdom

**Keywords:** microbial ecology, sea surface microlayer, SML, bacteria, cruise SO243, RV *Sonne*, peruvian upwelling region, transparent exopolymer particles

## Abstract

The sea surface microlayer (SML) is located at the air-sea interface, with microorganisms and organic matter in the SML influencing air-sea exchange processes. Yet understanding of the SML bacterial (bacterioneuston) community composition and assembly remains limited. Availability of organic matter, UV radiation and wind speed have previously been suggested to influence the community composition of bacterioneuston. Another mechanism potentially controlling bacterioneuston dynamics is bacterioplankton attached to gel-like particles that ascend through the water column into the SML. We analyzed the bacterial community composition, Transparent Exopolymer Particles (TEP) abundance and nutrient concentrations in the surface waters of the Peruvian upwelling region. The bacterioneuston and bacterioplankton communities were similar, suggesting a close spatial coupling. Four *Bacteroidetes* families were significantly enriched in the SML, two of them, the *Flavobacteriaceae* and *Cryomorphaceae*, were found to comprise the majority of SML-enriched operational taxonomic units (OTUs). The enrichment of these families was controlled by a variety of environmental factors. The SML-enriched bacterial families were negatively correlated with water temperature and wind speed in the SML and positively correlated with nutrient concentrations, salinity and TEP in the underlying water (ULW). The correlations with nutrient concentrations and salinity suggest that the enriched bacterial families were more abundant at the upwelling stations.

## Introduction

The sea surface microlayer (SML) constitutes the uppermost layer of the ocean, only 1–1000 μm thick, with unique chemical and biological properties that distinguish it from the underlying water (ULW) (Zhang et al., 2003; Liss and Duce, 2005). Due to the location at the air-sea interface, the SML can influence exchange processes across this boundary layer, such as air-sea gas exchange and the formation of sea spray aerosols (Liss and Duce, 2005; Wurl and Holmes, 2008; Aller et al., 2017; Engel et al., 2017).

The term ‘neuston’ describes the organisms in the SML and was first suggested by Naumann (1917). As in other marine ecosystems, bacterioneuston communities have important roles in SML functioning (Cunliffe et al., 2011). Bacterioneuston community composition of the SML has been analyzed and compared to the underlying water in different habitats with varying results, and has primarily focused on coastal waters and shelf seas, with limited study of the open ocean (Agogué et al., 2005a; Franklin et al., 2005; Cunliffe et al., 2009c). In the North Sea, a distinct bacterial community was found in the SML with *Vibrio* spp. and *Pseudoalteromonas* spp. dominating the bacterioneuston (Franklin et al., 2005). During an artificially induced phytoplankton bloom in a fjord mesocosm experiment, the most dominant denaturing gradient gel electrophoresis (DGGE) bands of the bacterioneuston consisted of two bacterial families: *Flavobacteriaceae* and *Alteromonadaceae* (Cunliffe et al., 2009c). Other studies have however, found little or no differences in the bacterial community composition of the SML and the ULW (Agogué et al., 2005a; Obernosterer et al., 2008). Difficulties in direct comparisons between studies can arise because of the different methods used to sample the SML, which result in varied sampling depths (Agogué et al., 2004; Cunliffe et al., 2009a, 2011).

Even less is known about the community control mechanisms in the SML and how the bacterial community assembles at the air-sea interface. The bacterioneuston community could be altered by differing wind conditions and radiation levels (Agogué et al., 2005b; Joux et al., 2006; Stolle et al., 2011; Rahlff et al., 2017), with high wind speeds inhibiting the formation of a distinct bacterioneuston community (Stolle et al., 2011; Rahlff et al., 2017). Wind speed and radiation levels refer to external controls, however, bacterioneuston community composition might also be influenced by internal factors such as nutrient availability and organic matter (OM) produced either in the SML or in the ULW (Azam et al., 1993; Judd et al., 2006; Nakajima et al., 2013).

One of the principal OM components consistently enriched in the SML are Transparent Exopolymer Particles (TEP) (Cunliffe et al., 2009b; Wurl et al., 2011; Engel and Galgani, 2016), which are rich in carbohydrates and form by the aggregation of dissolved precursors excreted by phytoplankton in the euphotic zone (Chin et al., 1998; Passow, 2000; Passow et al., 2001; Engel et al., 2004). Higher TEP formation rates in the SML, facilitated through wind shear and dilation of the surface water, have been proposed as one explanation for the observed enrichment in TEP (Wurl et al., 2011; Sun et al., 2018). Also, due to their natural positive buoyancy, when not ballasted by other particles sticking to them, TEP ascend through the water column and ultimately end up at the SML (Azetsu-Scott and Passow, 2004). A second possible pathway of TEP from the water column to the SML is by bubble scavenging (Zhou et al., 1998).

Next to rising bubbles, another potential transport mechanism for bacteria from the ULW to the SML could be ascending particles (Agogué et al., 2005a; Joux et al., 2006) or more specifically TEP (Azetsu-Scott and Passow, 2004). Bacteria readily attach to TEP in the water column (Schuster and Herndl, 1995; Mari and Kiorboe, 1996; Busch et al., 2017). TEP can serve as microbial hotspots and can be used directly as a substrate for bacterial degradation (Passow, 2002; Meiners et al., 2008; Taylor and Cunliffe, 2017), and as grazing protection for attached bacteria, e.g., by acting as an alternate food source for zooplankton (Malej and Harris, 1993; Passow and Alldredge, 1999; Dutz et al., 2005). TEP have also been suggested to serve as light protection for microorganisms in environments with high irradiation (Ortega-Retuerta et al., 2009).

This study focuses on understanding the bacterioneuston community composition as well as possible community control mechanisms. Therefore, we analyzed the composition of the bacterial community in the SML and ULW as well as various abiotic factors in addition to nutrients and TEP concentrations during a cruise in the Peruvian upwelling region.

## Materials and Methods

### Sampling

**FIGURE 1:**
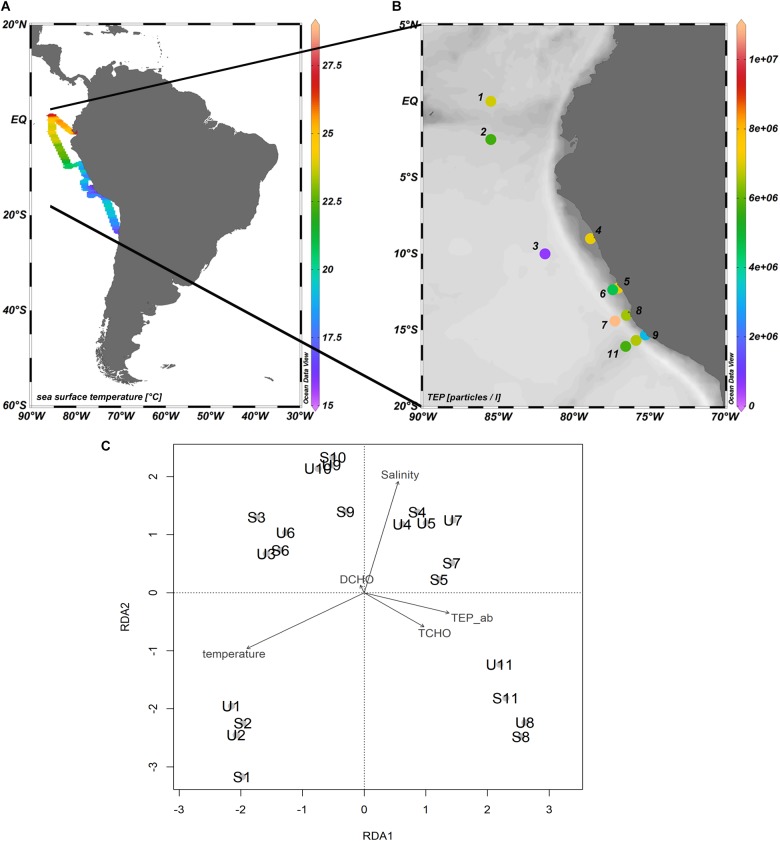
**(A)** Map of the cruise track of the RV *Sonne* in October 2015 during the SO243 cruise off the Peruvian coast. The color represents the sea surface temperature. The water temperature was measured below the ship at 2 m water depth and obtained from the DSHIP system onboard the RV *Sonne*. **(B)** Map of the stations sampled for SML and ULW. The colors represent the concentration of Transparent Exopolymer Particles (TEP) in the Sea Surface Microlayer in particles l^−1^. **(C)** RDA on OTU level of the all stations during SO243 according to their bacterial community composition with environmental factors. RD1 explains 54% of the variance within the data, RD2 explains 13%. U, ULW samples; S, SML samples.

Samples were collected during the SO243 cruise to the Peruvian upwelling region onboard the RV *Sonne* from the 4th to the 22nd of October 2015 (Figure 1A), with 11 stations sampled between 0°S and 15.7°S as well as 85.5°W and 75.3°W (Figure 1B). At each station, samples were collected from two depths: surface microlayer (SML) and underlying water (ULW) at 20 cm, except at station 1 where an ULW sample was taken at 10 cm. SML sampling was conducted using a glass plate sampler from a zodiac (Harvey, 1966; Cunliffe and Wurl, 2014). The 50 × 26 cm silicate glass plate had an effective sampling surface area of 2600 cm^2^ considering both sides. For sampling, the zodiac was located app. 0.5 nautical miles into the wind direction away from the research vessel to avoid contamination. The glass plate was immersed perpendicular to the sea surface and withdrawn at a controlled rate of ∼17 cm s^−1^. SML samples adhered to the plate and were collected in an acid cleaned and rinsed bottle using a Teflon wiper (Cunliffe and Wurl, 2014). All sampling equipment was cleaned with acid (10% HCl) and rinsed in MilliQ before sampling. In addition, all sampling devices were copiously rinsed with seawater from the respective depth when the sampling site was reached.

The ULW sample was collected with a LIMNOS water sampler (Hydro-bios) that had two acid cleaned (10% HCl) and MilliQ rinsed plastic bottles attached to it. A weight attached to the LIMNOS sampler ensured that the bottles could not float to the surface during sampling. Since long waves rather than short breaking waves were most prominent, the depth of the LIMNOS sampler could be adjusted manually by keeping a marker on the rope continuously at the surface during sampling. Sampling of the ULW took about 30 s in total, keeping depth variations to a minimum.

### Transparent Exopolymer Particles (TEP)

The abundance and area of TEP were measured microscopically (Engel, 2009). Depending on the concentration of TEP, 10–30 ml of the sample was filtered onto a 0.4 μm Nuclepore membrane (Whatman), and stained with 1 ml Alcian Blue solution (0.2 g l^−1^ w/v) for 3 s. Filters were subsequently mounted on Cytoclear^®^ slides and stored at −20°C. The Zeiss Axio Scope. A1 (Zeiss) and the AxioCam MRc (Zeiss) were used to analyze two filters per sample with 30 images each. The pictures were saved using AxioVision LE64 Rel. 4.8 (Zeiss) with a resolution of 1388 × 1040 pixels. All particles bigger than 0.2 μm^2^ were analyzed. Subsequent image analysis was conducted with ImageJ (Schneider et al., 2012). Two filters of 30 ml MilliQ water served as a blank.

### Total and Dissolved Combined Carbohydrates

20 ml samples were collected into combusted glass vials (8 h, 500°C) for the analysis of total and dissolved hydrolysable carbohydrates > 1 kDa (TCHO and DCHO, respectively). DCHO samples were filtered through a 0.45 μm Acrodisc^®^ syringe filter (Millipore) prior to filling the glass vials. Both TCHO and DCHO samples were stored at −20°C until analysis. Prior to analysis, samples were desalinated using membrane dialysis (1 kDa MWCO, Spectra Por) at 1°C for 5 h with a subsequent hydrolysis step for 20 h at 100°C with 0.8 M HCl final concentration and neutralization through acid evaporation (N_2_, for 5 h at 50°C). High performance anion exchange chromatography with pulsed amperometric detection (HPAEC-PAD) was applied on a Dionex ECS 3000 ion chromatography system (Engel and Händel, 2011) to measure the samples. Two replicates per sample were analyzed.

### Bacterial Numbers

Bacterial numbers were determined using flow cytometry from a 4 ml sample that was fixed with 200 μl glutaraldehyde (GDA, 1% final concentration). Samples were stored at −20°C for at most 2.5 months until analysis and were stained with SYBR Green I (Molecular Probes) prior to quantification using a flow cytometer equipped with a 488 nm laser (Becton & Dickinson FACScalibur). A plot of side scatter (SSC) vs. green fluorescence (FL1) was used to detect the unique signature of the bacterial cells. The internal standard consisted of yellow-green latex beads (Polysciences, 0.5 μm).

### DNA Extraction and Bacterial 16S rRNA Gene Sequencing

For DNA extraction, 400 ml of sample was filtered onto a Durapore membrane (Millipore, 47 mm, 0.2 μm) and stored at −80°C. Prior to DNA extraction, tubes containing the filters were immersed in liquid nitrogen and the filter was crushed with a pestle to make the cell membrane accessible for the DNA extraction buffers. DNA was extracted using a modified protocol from Zhou et al. (1996) by Wietz et al. (2015) and included bead-beating, phenol-chloroform-isoamyl alcohol purification, isopropanol precipitation and ethanol washing. An additional protein-removal step by salting was added. DNA concentrations in the sample were measured with a Qubit Fluorometer (Life Technologies) and stored at −20°C.

Bacterial 16S rRNA gene amplicons were sequenced on a MiSeq platform (Illumina). Primers used to PCR amplify the region of interest were 341F and 805R (Herlemann et al., 2011) recommended in the MiSeq protocol (Illumina). The samples were prepared according to the manufacturer’s instructions with modifications. Three PCR steps were used to amplify the region of interest: one PCR amplifying the 16S rRNA gene amplicons, one ligating the amplicons to the adapters and the third to add the index primers to the adapters with 20, 10, and 8 cycles, respectively. The PCR cycling conditions were the same for all three steps: initial denaturation at 95°C for 3 min, cycles with denaturation at 95°C for 30 s, annealing at 55°C for 30 s and elongation at 72°C for 30 s. A final elongation was carried out at 72°C for 5 min. DNA was purified using the AMPure XP bead purification kit according to the manufacturer’s instructions (Beckmann Coulter).

### Bioinformatics and Statistical Analyses

Sequences were quality-trimmed using mothur v 1.35.1 (Schloss et al., 2009). Sequences shorter than 400 bp or longer than 467 bp were removed, as well as sequences with more than 8 homopolymers and any ambiguous bases. Sequences were aligned with the SILVA alignment as reference (Quast et al., 2013). After discarding sequences that aligned outside the majority of the dataset and removing chimeras, the Greengenes database (DeSantis et al., 2006) was used to classify the sequences which were deposited at the European Nucleotide Archive (ENA accession number PRJEB22038).

Statistical analyses were conducted in R (R Core Team, 2014). Ocean Data View was used to compute the map of the cruise (Schlitzer, 2013). The significance of enrichment/depletion of the abundant families was obtained by testing the enrichment factor (EF) of the respective families against an EF = 1 (signifying no enrichment or depletion) with a *t*-test. The EF was used to compare the concentration of substance A in the SML compared to the ULW and was calculated using the following formula:

(1) $$\mathrm{EF}={\left[A\right]}_{\text{SML}}/{\left[A\right]}_{\text{ULW}}$$

Where [A] represents the concentration of a certain substance in the SML or ULW (World Health Organization, 1995). An EF > 1 indicates enrichment, whereas an EF < 1 indicates depletion of a certain substance in the SML compared to the ULW.

The correlation of the enriched families with environmental factors was calculated using a Spearman rank correlation because the bacterial family abundances were not normally distributed.

### Data Obtained From the Ship

Abiotic data, such as irradiance, wind speed and water temperature was obtained from the DSHIP system on the RV *Sonne*. Water temperature was measured at a depth of 2 m, wind speed was measured 35 m above the water surface.

## Results

### Transparent Exopolymer Particles and Combined Carbohydrates

Figure 1 shows the sea surface temperature (Figure 1A) and TEP abundance (Figure 1B) during the cruise. TEP were found in abundances ranging from 5.78 × 10^5^ to 1.09 × 10^7^ particles l^−1^ (average of 5.89 × 10^6^ ± 2.47 × 10^6^ particles l^−1^) in the SML compared to 6.58 × 10^5^ to 1.18 × 10^7^ particles l^−1^ (average of 4.43 × 10^6^ ± 3.37 × 10^6^ particles l^−1^) in the ULW. The highest abundances of TEP were found in the to upwelling, close to the Peruvian coast between stations 4–8 (Figure 1B). TCHO concentrations varied between 543 and 1204 nmol l^−1^ in the SML and 463 and 1495 nmol l^−1^ in the ULW (Supplementary Figure S2), whereas DCHO concentrations ranged from 352 to 696 nmol l^−1^ in the SML and 228 to 1200 nmol l^−1^ in the ULW (Supplementary Figure S3).

### Bacterial Abundance and Community Composition

The total bacterial numbers determined by flow cytometry were not significantly different between the SML and the ULW (*p* = 0.89, *t*-test) and were strongly correlated (rho = 0.94, *p* < 0.05). Bacterial abundances ranged from 4.64 × 10^5^ to 1.75 × 10^6^ cells ml^−1^ (average of 8.89 × 10^5^ ± 4.31 × 10^5^ cells ml^−1^) in the SML and 4.42 × 10^5^ to 1.89 × 10^6^ cells ml^−1^ (average of 9.18 × 10^5^ ± 4.65 × 10^5^ cells ml^−1^) in the ULW, respectively (Supplementary Table S3). EFs varied between 0.80 and 1.28 with an average of 0.98 ± 0.13.

The bacterial community structure of the SML and the ULW was assessed based on 16S rRNA gene high-throughput sequencing. Figure 1C shows a RDA plot of all stations during the cruise, highlighting the general similarity of the bacterial communities between the SML and the ULW at almost all stations. At some stations (S1, 3, 4, 5, 7, 9) one or both of the depths sampled was more similar to another station than to the corresponding depth at the same station. No clear trend could be observed between the location of the stations and the similarity of the bacterial communities (e.g., distance from the coast). The bacterial diversity was similar between SML and ULW communities with no clear trend of either depth having a higher diversity (Supplementary Table S2).

### Bacterial Families Enriched in the SML

**FIGURE 2:**
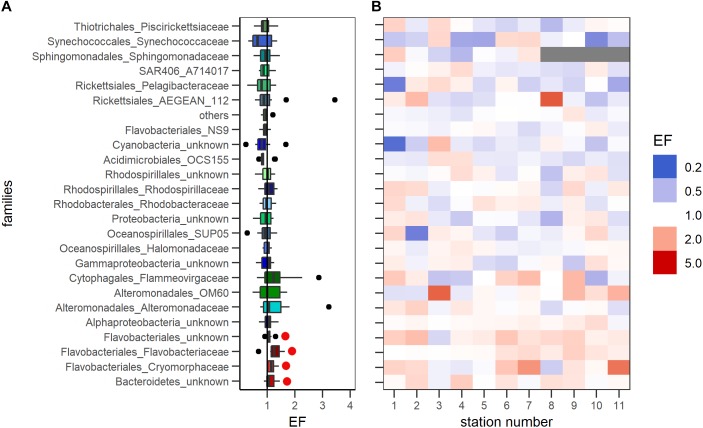
**|** Enrichment of bacterial families that make up at least 1% of the total bacterial community in at least two stations. **(A)** Enrichments during the whole cruise (11 stations) were combined. Red, Bacterial families that were significantly enriched in the SML. blue/green, bacterial families that showed no significant changes with depth. Black line indicates no change between depths (EF = 1). Black circles, outliers. Red dots mark significant enrichment of bacterial families throughout the cruise. **(B)** Enrichment of families according to each individual station. Blue indicates a depletion, white no change and red an enrichment of a certain bacterial family at the respective station. An EF of 0.2 indicates a five-fold decrease, an EF of 5 a five-fold increase of a certain bacterial species in the SML.

Twenty four families were identified that made up at least 1% each of the total community at two stations or more considering both depths (Supplementary Table S1). These families were considered to be important for the community composition and analyzed in further detail. All other families were summarized as ‘others.’ None of the bacterial families showed a significant depletion in the SML compared to the ULW (*t*-test against an EF = 1, *p* < 0.05) (Figure 2A). Four families (unknown *Flavobacteriales*, *Flavobacteriaceae*, *Cryomorphaceae*, unknown *Bacteroidetes*) were however, significantly enriched in the SML compared to the ULW throughout the cruise (*t*-test against an EF = 1, *p* < 0.05) (Figure 2A), with all members belonging to the phylum *Bacteroidetes.* Figure 2B shows that the four families were consistently enriched at almost all stations analyzed.

**FIGURE 3:**
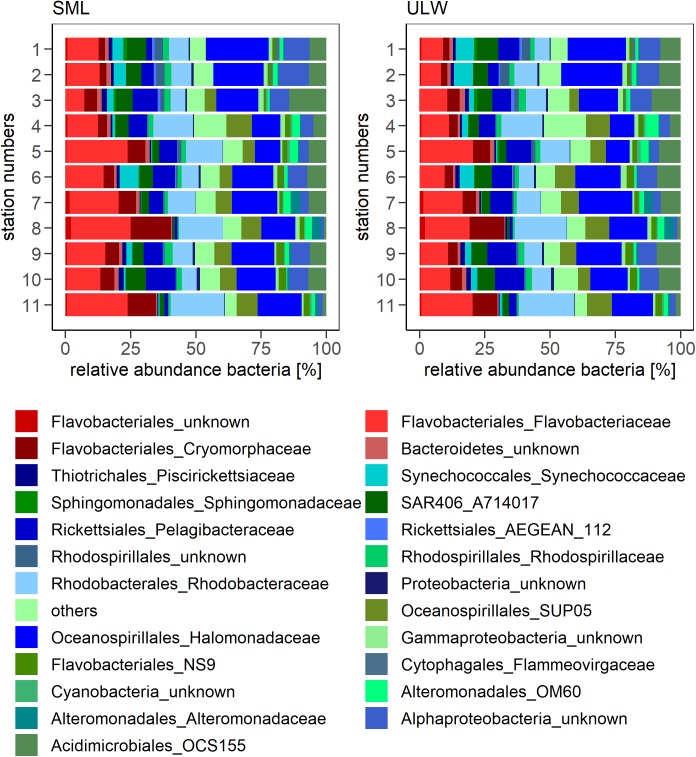
Bacterial community composition at all stations given as relative abundances at family level. Families that make up > 1% in at least two stations are shown. Red, significantly enriched families in the SML. Blue/green, bacterial families showing no differences between SML and ULW.

**FIGURE 4:**
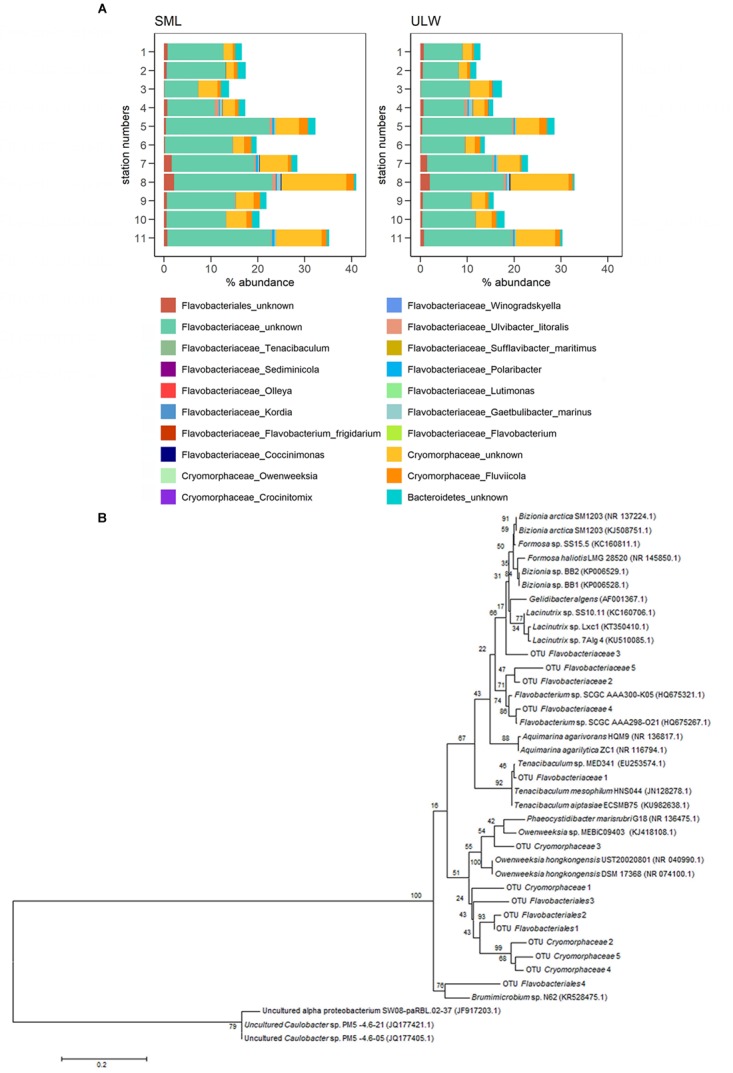
**(A)** Community composition of enriched bacterial families at species level. **(B)** Phylogenetic tree of the most abundant OTUs of unknown *Flavobacteriales* family, *Flavobacteriaceae*, and *Cryomorphaceae*. The closest relatives identified at the genus level were included in the tree, Alphaproteobacteria were used as an outgroup. The sequences were aligned with MUSCLE. The tree was computed with the maximum likelihood method. The scale bar represents 20% sequence divergence. Numbers at nodes represent bootstrap values (*n* = 100).

The relative abundances of all four families that were significantly enriched in the SML (Figure 2A) were pooled to calculate the overall relative abundance of SML enriched bacterial families. Figure 3 shows the community composition across all stations in the SML and ULW. The enriched families made up 24 ± 8% in the SML and 20 ± 7% in the ULW across all stations (Figure 4A). While the means of the relative abundance of the combined enriched families in the SML and ULW did not differ significantly (*t*-test, *p* = 0.25), the EFs during the cruise were significantly different from 1 (*t*-test against an EF = 1, *p* < 0.05). Out of the four SML enriched bacterial families, *Flavobacteriaceae*, and *Cryomorphaceae* were dominating (Figure 4A). The composition of the enriched bacterial families was similar between SML and ULW, the overall relative abundance was lower in the ULW though (Figure 4A). The five most abundant OTUs of *Flavobacteriaceae*, *Cryomorphaceae*, and unknown *Flavobacteriales* were further analyzed. Figure 4B shows their closest neighbors. The majority of unknown *Flavobacteriales* OTUs cluster rather closely with the *Cryomorphaceae*. Figure 3 shows that the community differences were higher between stations than between SML and ULW, which showed similar bacterial communities. The most abundant families at both depths were *Flavobacteriaceae*, *Rhodobacteraceae* as well as *Halomonadales* at some stations.

### Control of SML-Enriched Families

The relationship of the pooled enriched bacterial families with abiotic and biotic factors (nutrients, salinity, radiation, wind speed, water temperature) was investigated to elucidate the driving factors of bacterial community enrichment in the SML. The SML enriched bacterial families (as calculated in the previous section) were positively correlated with nutrients such as phosphate (PO_4_), nitrate (NO_3_), silicate (SiO_2_), and salinity and were negatively correlated with seawater temperature (rho_SML_ = −0.93, rho_ULW_ = −0.91, respectively, *p* < 0.05) and wind speed (rho = −0.64, *p* < 0.05) (Table 1). The enrichment of the pooled SML enriched families did not correlate significantly with wind speed. This means that high wind speeds were co-occurring with low abundances of SML enriched families not only in the SML, but also in the ULW, thus not affecting the EF. No correlation was found between enriched bacterial families and instantaneous irradiation intensity neither in the SML nor in the ULW. The SML enriched bacterial families showed a significant positive correlation with both TEP abundance and area in the ULW (rho_abundance_ = 0.80 and rho_area_ = 0.80, respectively, *p* < 0.05), and were also positively correlated with TCHO in the ULW (rho = 0.71, *p* < 0.05), but showed no correlation with DCHO.

**Table 1:** Correlations of enriched bacterial families in the SML with different biotic and abiotic parameters in the SML and the ULW.

Parameter	rho_SML_	rho_ULW_
TEP	–	0.80
TEP area	–	0.80
TCHO	–	0.71
PO_4_	0.84	0.85
NO_3_	0.73	0.75
NO_2_	–	–
SiO_2_	0.65	0.72
Salinity	0.77	0.73
Radiation	–	–
Wind speed	−0.64	–
Water temperature	−0.93	−0.91

*Only significant correlations (p < 0.05) are displayed.*

## Discussion

The overall bacterial community composition was similar between SML and ULW throughout the cruise (Figure 1C), which is in accordance with some previous studies (Agogué et al., 2005a; Stolle et al., 2011; Taylor and Cunliffe, 2014). The use of a metal screen sampler might potentially have led to higher SML enrichments as was found previously by Agogué et al. (2004). The same authors showed in a later study that the different sampling methods (metal screen, glass plate sampler and membrane) all retrieved the same bacterial species (Agogué et al., 2005a), thus we are confident that our samples are a good representation of the SML bacterial community during the cruise, potentially rather underestimating the enrichment of bacteria in the SML. Figure 1C shows that the bacterial communities cluster rather by station than by depth. A strong correlation (rho = 0.94, *p* < 0.05) between bacterial numbers in the SML and ULW was observed, suggesting a close coupling between the two environments. The concentration of TEP in the SML on the other hand seemed to be less determined by the concentration in the ULW, but rather by the station location as discussed previously in detail by Zäncker et al. (2017).

The bacterial community in the SML as well as the ULW was dominated by *Flavobacteriaceae*, *Cryomorphaceae* (both members of *Bacteroidetes*), *Rhodobacteraceae*, and *Halomonadaceae*. Members of *Bacteroidetes* have been found to be abundant in the SML in the Baltic Sea in a previous study (Stolle et al., 2011), corroborating our findings. In contrast to a previous study (Franklin et al., 2005), the bacterial diversity did not differ significantly between the SML and the ULW during our cruise. While the bulk of the bacterial community did not show clear differences between the SML and ULW, four out of the 24 abundant families (>1% relative abundance at two or more stations) that were all members of the *Bacteroidetes*, showed a significant enrichment in the SML considering all stations. While the enrichment (Figure 2A) was not high (average EF of 1.22 ± 0.18), it was consistent throughout the cruise (Figure 2B). The positive correlations of the abundance of the enriched bacterial community with nutrient concentrations and salinity and the negative correlation with water temperature indicate that these bacteria were more abundant at upwelling stations (S7–11) compared to the rest of the stations (S1–S6) (Supplementary Figure S1).

Additional factors possibly influencing the bacterial community composition were analyzed. Several factors have been proposed to control the bacterial diversity in the SML *in situ*, including meteorological conditions, exposure to UV radiation, aerosol deposition and organic matter availability (Carlucci et al., 1985; Agogué et al., 2005a; Stolle et al., 2010, 2011; Nakajima et al., 2013; Astrahan et al., 2016). During this study, a negative correlation of the SML abundance of enriched bacterial families with wind speed was found, which corroborates earlier findings that high wind speeds disrupt the SML and seem to prevent bacterial enrichment in the SML of the Baltic Sea and during a wind wave tunnel experiment (Rahlff et al., 2017).

No correlation was found between the abundance of enriched bacterial families and instantaneous irradiation intensity suggesting UV radiation was not the main factor driving the bacterioneuston community composition. However, the correlation was conducted using instantaneous irradiation only, the irradiation history was not considered and thus an effect of the irradiation history cannot be excluded. In a laboratory study for example, bacteria responded to irradiation exposure after 1–2 h (Joux et al., 1999). Furthermore, the difference of UV radiation intensity between the SML and the ULW was not measured. At high light intensities the difference between the light entering the water and the light that reaches 1 m can be high (Fleischmann, 1989). Considering that light intensity exponentially decreases in the water column, it seems reasonable to expect differences in the light intensities between the SML and the ULW at 20 cm, though.

Next to *in situ* growth of bacteria in the SML, the bacterionneuston community composition might also be influenced by factors influencing the upward transport of bacteria such as bubbles and positively buoyant particles, particularly TEP (Azetsu-Scott and Passow, 2004; Agogué et al., 2005a; Joux et al., 2006). Among the most abundant enriched families were *Flavobacteriales*, which have been shown previously to be involved in polysaccharide degradation (Buchan et al., 2014; Teeling et al., 2016). A direct link with TEP and TEP precursors, such as polysaccharides produced by phytoplankton, has also been demonstrated for *Rhodobacterales*, *Flavobacteriales*, and *Alteromonadales* (Buchan et al., 2014; Taylor et al., 2014; Teeling et al., 2016; Taylor and Cunliffe, 2017).

The correlations between enriched families and TEP found in the present study suggest that enriched families might be associated with TEP. Since TEP are positively buoyant (Azetsu-Scott and Passow, 2004), it seems plausible that TEP can act as a transport mechanism for the bacteria attached from the ULW to the SML, which might be one way for bacteria to be enriched in the SML. The percentage of the total bacterial community that is attached to TEP has been previously observed to be 0.5–20% (Mari and Kiorboe, 1996; Busch et al., 2017). Assuming that most members of the four enriched families in the SML and ULW live attached to TEP, the previously published numbers are in agreement with the relative abundance of enriched families in the SML and ULW (on average 24 ± 8% in the SML and 20 ± 7% in the ULW) found during this study. At the same time, this would mean that the potential importance of TEP is limited to only part of the total bacterial community. The similar community structure of the SML and ULW throughout the cruise furthermore suggests a limited influence. Further studies would be needed to analyze the exact effect of TEP on the bacterionneuston community composition including whether attachment to TEP enhances growth *in situ* in the SML, or whether it enhances the transport up from the ULW. The missing correlation of enriched bacterial families and TEP in the SML might be caused by wind speed affecting the bacterionneuston community (as discussed above), but not TEP in the SML (as discussed in Zäncker et al., 2017).

Our study reports the bacterial community composition in the SML and ULW off the Peruvian coast. In addition, it discusses potential influencing factors of the bacterionneuston community composition such as nutrients, wind speed, and interactions with organic matter. Future studies are necessary to elucidate the potential impact of upward transport of bacteria into the SML further.

## Author Contributions

All authors listed have made a substantial, direct and intellectual contribution to the work, and approved it for publication.

## Conflict of Interest Statement

The authors declare that the research was conducted in the absence of any commercial or financial relationships that could be construed as a potential conflict of interest.
