# Supplementary material for: Bacterial Community Composition in the Sea Surface Microlayer Off the Peruvian Coast
Source: Front Microbiol. 2018 Nov 15;9:2699. doi: 10.3389/fmicb.2018.02699 (PMC6249803; doi:10.3389/fmicb.2018.02699)
Supplement: Supplementary file 2 [file Data_Sheet_2.PDF]

Supplementary Material

Bacterial Community Composition in the Sea Surface Microlayer off the Peruvian Coast

Birthe Zäncker[1*], Michael Cunliffe[2,3], Anja Engel[1]

[1]GEOMAR – Helmholtz Centre for Ocean Research Kiel, Kiel, Germany

[2]Marine Biological Association of the UK, The Laboratory, Citadel Hill, Plymouth, UK

[3]Marine Biology and Ecology Research Centre, School of Biological and Marine Sciences, Plymouth University, Plymouth, UK

***Correspondence:**

Birthe Zäncker

bzaencker@posteo.de

1 Supplementary Figure

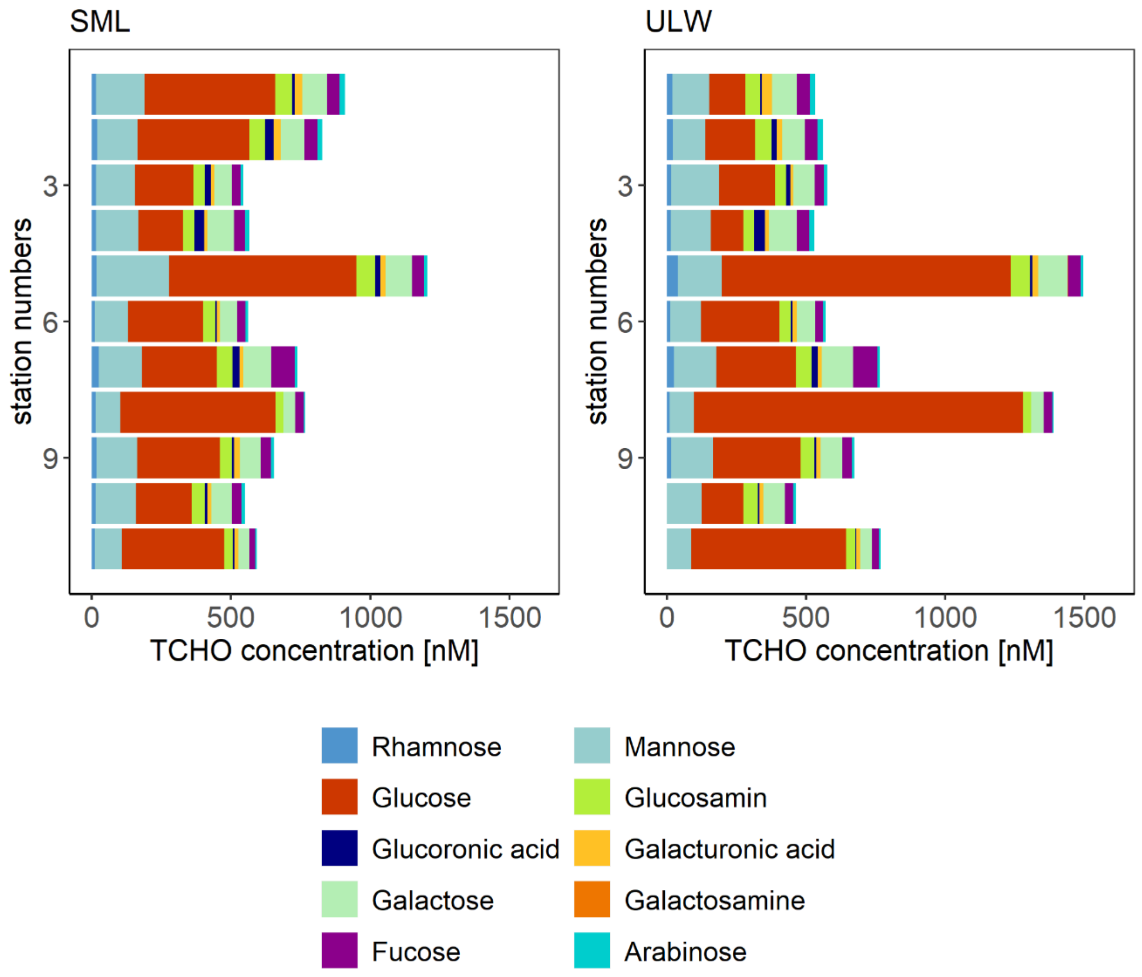

Figure S2: Composition of total combined carbohydrates (TCHO) across stations (on the y-axis) in nM. Gluconic acid and muramic acid could not be detected in any of the stations and are thus not included in the graph.
