# Supplementary material for: Bacterial Community Composition in the Sea Surface Microlayer Off the Peruvian Coast
Source: Front Microbiol. 2018 Nov 15;9:2699. doi: 10.3389/fmicb.2018.02699 (PMC6249803; doi:10.3389/fmicb.2018.02699)
Supplement: Supplementary file 4 [file Table_1.DOCX]

Supplementary Material

# Bacterial Community Composition in the Sea Surface Microlayer off the Peruvian Coast

**Birthe Zäncker^1*^, Michael Cunliffe^2,3^, Anja Engel^1^**

^1^GEOMAR – Helmholtz Centre for Ocean Research Kiel, Kiel, Germany

^2^Marine Biological Association of the UK, The Laboratory, Citadel Hill, Plymouth, UK

^3^Marine Biology and Ecology Research Centre, School of Biological and Marine Sciences, Plymouth University, Plymouth, UK

***Correspondence:**

Birthe Zäncker

bzaencker@posteo.de

# Supplementary Table

Table S1: Overview of all detected bacterial genera across all stations and depths.

| **family** | **S1 SML** | **S1 ULW** | **S2 SML** | **S2 ULW** | **S3 SML** | **S3 ULW** | **S4 SML** | **S4 ULW** | **S5 SML** | **S5 ULW** | **S6 SML** | **S6 ULW** | **S7 SML** | **S7 ULW** | **S8 SML** | **S8 ULW** | **S9 SML** | **S9 ULW** | **S10 SML** | **S10 ULW** | **S11 SML** | **S11 ULW** |
| --- | --- | --- | --- | --- | --- | --- | --- | --- | --- | --- | --- | --- | --- | --- | --- | --- | --- | --- | --- | --- | --- | --- |
| Acidobacteria_Subgroup_26_Subgroup_26_Subgroup_26 | 0.00 | 0.00 | 0.00 | 0.00 | 0.00 | 0.00 | 0.00 | 0.00 | 0.00 | 0.00 | 0.00 | 0.00 | 0.00 | 0.00 | 0.01 | 0.00 | 0.00 | 0.00 | 0.00 | 0.00 | 0.00 | 0.01 |
| Actinobacteria | 0.01 | 0.03 | 0.01 | 0.02 | 0.13 | 0.14 | 0.00 | 0.01 | 0.05 | 0.03 | 0.02 | 0.02 | 0.02 | 0.01 | 0.02 | 0.01 | 0.08 | 0.09 | 0.02 | 0.13 | 0.00 | 0.02 |
| Actinobacteria_Acidimicrobiia_Acidimicrobiales | 0.00 | 0.00 | 0.00 | 0.00 | 0.00 | 0.00 | 0.00 | 0.00 | 0.00 | 0.00 | 0.00 | 0.00 | 0.00 | 0.00 | 0.00 | 0.00 | 0.00 | 0.00 | 0.00 | 0.00 | 0.00 | 0.01 |
| Actinobacteria_Acidimicrobiia_Acidimicrobiales_Acidimicrobiaceae | 0.13 | 0.12 | 0.13 | 0.15 | 0.02 | 0.03 | 0.24 | 1.23 | 0.37 | 0.38 | 0.04 | 0.04 | 0.37 | 0.34 | 0.12 | 0.26 | 0.03 | 0.02 | 0.01 | 0.02 | 0.03 | 0.03 |
| Actinobacteria_Acidimicrobiia_Acidimicrobiales_OM1_clade | 6.44 | 7.69 | 6.37 | 8.16 | 13.46 | 10.81 | 5.07 | 3.93 | 6.94 | 7.99 | 7.32 | 8.75 | 6.72 | 7.42 | 0.80 | 1.08 | 6.36 | 8.95 | 6.61 | 8.33 | 1.37 | 1.82 |
| Actinobacteria_Acidimicrobiia_Acidimicrobiales_Sva0996_marine_group | 0.45 | 0.36 | 0.51 | 0.56 | 0.98 | 1.44 | 0.35 | 0.54 | 0.28 | 0.24 | 1.29 | 1.11 | 0.10 | 0.10 | 0.16 | 0.16 | 0.40 | 0.25 | 0.25 | 0.22 | 0.11 | 0.10 |
| Actinobacteria_Actinobacteria_Bifidobacteriales_Bifidobacteriaceae | 0.01 | 0.02 | 0.00 | 0.01 | 0.01 | 0.01 | 0.01 | 0.02 | 0.01 | 0.01 | 0.01 | 0.02 | 0.00 | 0.00 | 0.00 | 0.01 | 0.00 | 0.01 | 0.00 | 0.00 | 0.01 | 0.00 |
| Actinobacteria_Actinobacteria_Corynebacteriales_Corynebacteriaceae | 0.00 | 0.00 | 0.00 | 0.00 | 0.00 | 0.00 | 0.00 | 0.00 | 0.00 | 0.00 | 0.00 | 0.00 | 0.00 | 0.00 | 0.00 | 0.00 | 0.00 | 0.00 | 0.00 | 0.00 | 0.00 | 0.00 |
| Actinobacteria_Actinobacteria_Corynebacteriales_Nocardiaceae | 0.00 | 0.00 | 0.00 | 0.00 | 0.00 | 0.00 | 0.00 | 0.00 | 0.00 | 0.00 | 0.00 | 0.00 | 0.00 | 0.00 | 0.00 | 0.00 | 0.00 | 0.00 | 0.00 | 0.00 | 0.00 | 0.00 |
| Actinobacteria_Actinobacteria_Micrococcales_Micrococcaceae | 0.01 | 0.00 | 0.00 | 0.00 | 0.00 | 0.02 | 0.00 | 0.00 | 0.00 | 0.00 | 0.00 | 0.00 | 0.00 | 0.00 | 0.00 | 0.00 | 0.01 | 0.00 | 0.01 | 0.00 | 0.00 | 0.00 |
| Actinobacteria_Actinobacteria_PeM15 | 0.01 | 0.00 | 0.01 | 0.02 | 0.02 | 0.02 | 0.13 | 0.28 | 0.08 | 0.12 | 0.01 | 0.01 | 0.10 | 0.04 | 0.03 | 0.02 | 0.01 | 0.03 | 0.00 | 0.02 | 0.01 | 0.01 |
| Actinobacteria_Actinobacteria_Propionibacteriales_Propionibacteriaceae | 0.00 | 0.00 | 0.00 | 0.00 | 0.00 | 0.00 | 0.00 | 0.01 | 0.00 | 0.00 | 0.00 | 0.00 | 0.00 | 0.00 | 0.00 | 0.00 | 0.00 | 0.00 | 0.00 | 0.00 | 0.00 | 0.00 |
| Actinobacteria_Thermoleophilia | 0.00 | 0.00 | 0.00 | 0.00 | 0.00 | 0.00 | 0.00 | 0.01 | 0.00 | 0.00 | 0.00 | 0.00 | 0.00 | 0.00 | 0.00 | 0.00 | 0.00 | 0.00 | 0.00 | 0.00 | 0.00 | 0.00 |
| Bacteria_unknown | 0.00 | 0.00 | 0.01 | 0.00 | 0.00 | 0.00 | 0.01 | 0.01 | 0.00 | 0.00 | 0.00 | 0.00 | 0.00 | 0.00 | 0.00 | 0.00 | 0.00 | 0.00 | 0.00 | 0.00 | 0.02 | 0.00 |
| Bacteria_unknown | 0.28 | 0.42 | 0.26 | 0.37 | 0.48 | 0.34 | 0.85 | 1.03 | 0.24 | 0.46 | 0.25 | 0.30 | 0.11 | 0.19 | 0.09 | 0.07 | 0.18 | 0.25 | 0.35 | 0.28 | 0.08 | 0.11 |
| Bacteria_unknown | 0.07 | 0.11 | 0.03 | 0.06 | 2.99 | 1.39 | 1.02 | 0.75 | 1.19 | 2.71 | 1.34 | 2.05 | 0.29 | 0.77 | 0.11 | 0.20 | 0.79 | 1.94 | 0.78 | 0.63 | 0.55 | 1.32 |
| Bacteroidetes | 0.14 | 0.22 | 0.29 | 0.31 | 0.14 | 0.13 | 0.19 | 0.22 | 0.08 | 0.09 | 0.22 | 0.19 | 0.11 | 0.11 | 0.07 | 0.05 | 0.19 | 0.15 | 0.64 | 0.67 | 0.08 | 0.10 |
| Bacteroidetes_Bacteroidetes_BD2-2 | 0.00 | 0.00 | 0.00 | 0.00 | 0.00 | 0.00 | 0.00 | 0.00 | 0.00 | 0.00 | 0.00 | 0.00 | 0.00 | 0.00 | 0.00 | 0.00 | 0.00 | 0.00 | 0.00 | 0.00 | 0.01 | 0.00 |
| Bacteroidetes_Bacteroidetes_VC2.1_Bac22 | 0.00 | 0.00 | 0.00 | 0.00 | 0.00 | 0.00 | 0.07 | 0.04 | 0.02 | 0.01 | 0.01 | 0.01 | 0.01 | 0.00 | 0.09 | 0.07 | 0.00 | 0.00 | 0.01 | 0.02 | 0.03 | 0.02 |
| Bacteroidetes_Bacteroidia_Bacteroidales_Bacteroidales_S24-7_group | 0.01 | 0.01 | 0.00 | 0.02 | 0.01 | 0.00 | 0.01 | 0.00 | 0.01 | 0.02 | 0.01 | 0.00 | 0.02 | 0.02 | 0.00 | 0.02 | 0.01 | 0.02 | 0.02 | 0.01 | 0.01 | 0.00 |
| Bacteroidetes_Bacteroidia_Bacteroidales_Marinilabiaceae | 0.00 | 0.00 | 0.00 | 0.00 | 0.00 | 0.00 | 0.00 | 0.00 | 0.00 | 0.00 | 0.00 | 0.00 | 0.00 | 0.00 | 0.00 | 0.00 | 0.00 | 0.00 | 0.00 | 0.00 | 0.00 | 0.00 |
| Bacteroidetes_Bacteroidia_Bacteroidales_Prevotellaceae | 0.01 | 0.02 | 0.01 | 0.02 | 0.01 | 0.00 | 0.01 | 0.02 | 0.01 | 0.02 | 0.00 | 0.00 | 0.01 | 0.03 | 0.01 | 0.02 | 0.00 | 0.00 | 0.00 | 0.01 | 0.00 | 0.01 |
| Bacteroidetes_Bacteroidia_Bacteroidales_Rikenellaceae | 0.00 | 0.01 | 0.00 | 0.01 | 0.00 | 0.00 | 0.00 | 0.00 | 0.00 | 0.00 | 0.00 | 0.00 | 0.00 | 0.00 | 0.00 | 0.00 | 0.00 | 0.00 | 0.00 | 0.00 | 0.00 | 0.00 |
| Bacteroidetes_Bacteroidia_Bacteroidia_Incertae_Sedis_Prolixibacteraceae | 0.00 | 0.00 | 0.00 | 0.00 | 0.00 | 0.00 | 0.00 | 0.00 | 0.00 | 0.00 | 0.00 | 0.00 | 0.00 | 0.00 | 0.01 | 0.01 | 0.00 | 0.00 | 0.00 | 0.00 | 0.00 | 0.00 |
| Bacteroidetes_Cytophagia_Cytophagales | 0.00 | 0.01 | 0.00 | 0.00 | 0.04 | 0.00 | 0.00 | 0.00 | 0.01 | 0.02 | 0.00 | 0.00 | 0.00 | 0.01 | 0.00 | 0.00 | 0.00 | 0.00 | 0.00 | 0.00 | 0.00 | 0.00 |
| Bacteroidetes_Cytophagia_Cytophagales | 0.00 | 0.00 | 0.00 | 0.00 | 0.00 | 0.00 | 0.01 | 0.01 | 0.00 | 0.02 | 0.01 | 0.00 | 0.01 | 0.06 | 0.01 | 0.01 | 0.00 | 0.00 | 0.00 | 0.01 | 0.00 | 0.00 |
| Bacteroidetes_Cytophagia_Cytophagales_Cyclobacteriaceae | 0.01 | 0.01 | 0.00 | 0.01 | 0.00 | 0.00 | 0.00 | 0.00 | 0.00 | 0.00 | 0.00 | 0.00 | 0.00 | 0.00 | 0.00 | 0.00 | 0.00 | 0.00 | 0.00 | 0.00 | 0.00 | 0.00 |
| Bacteroidetes_Cytophagia_Cytophagales_Cytophagaceae | 0.00 | 0.00 | 0.00 | 0.00 | 0.00 | 0.00 | 0.00 | 0.00 | 0.00 | 0.00 | 0.00 | 0.00 | 0.00 | 0.01 | 0.00 | 0.00 | 0.00 | 0.00 | 0.00 | 0.01 | 0.00 | 0.00 |
| Bacteroidetes_Cytophagia_Cytophagales_Flammeovirgaceae | 1.56 | 1.17 | 0.68 | 0.49 | 0.18 | 0.21 | 0.02 | 0.05 | 0.12 | 0.14 | 0.30 | 0.18 | 0.11 | 0.15 | 0.07 | 0.12 | 0.32 | 0.24 | 0.12 | 0.13 | 0.07 | 0.03 |
| Bacteroidetes_Cytophagia_Order_II_Rhodothermaceae | 0.00 | 0.00 | 0.00 | 0.00 | 0.00 | 0.00 | 0.01 | 0.00 | 0.00 | 0.00 | 0.00 | 0.00 | 0.00 | 0.00 | 0.01 | 0.01 | 0.00 | 0.00 | 0.00 | 0.00 | 0.00 | 0.00 |
| Bacteroidetes_Flavobacteriia_Flavobacteriales | 0.19 | 0.22 | 0.21 | 0.14 | 0.12 | 0.17 | 0.04 | 0.05 | 0.05 | 0.08 | 0.07 | 0.09 | 0.01 | 0.01 | 0.04 | 0.04 | 0.07 | 0.07 | 0.03 | 0.04 | 0.01 | 0.01 |
| Bacteroidetes_Flavobacteriia_Flavobacteriales_Cryomorphaceae | 2.20 | 2.04 | 2.52 | 2.09 | 2.50 | 2.67 | 3.49 | 3.18 | 5.73 | 5.12 | 2.75 | 2.11 | 6.79 | 5.29 | 15.11 | 13.16 | 4.53 | 3.21 | 4.73 | 3.75 | 10.63 | 9.40 |
| Bacteroidetes_Flavobacteriia_Flavobacteriales_Flavobacteriaceae | 11.89 | 8.18 | 12.44 | 7.66 | 7.41 | 10.25 | 11.16 | 10.45 | 23.58 | 19.40 | 13.78 | 9.47 | 18.52 | 15.06 | 22.92 | 17.02 | 14.54 | 10.05 | 12.55 | 11.33 | 23.26 | 19.36 |
| Bacteroidetes_Flavobacteriia_Flavobacteriales_NS7_marine_group | 1.10 | 0.80 | 1.04 | 0.75 | 0.91 | 1.05 | 1.03 | 0.62 | 1.18 | 1.09 | 0.56 | 0.45 | 0.94 | 1.00 | 0.34 | 0.26 | 0.79 | 0.71 | 1.11 | 0.94 | 0.27 | 0.18 |
| Bacteroidetes_Flavobacteriia_Flavobacteriales_NS9_marine_group | 2.21 | 2.40 | 1.98 | 2.22 | 3.41 | 3.54 | 2.93 | 2.83 | 3.35 | 3.29 | 4.05 | 3.86 | 2.91 | 2.81 | 3.88 | 4.09 | 4.13 | 3.64 | 3.96 | 3.78 | 3.27 | 3.50 |
| Bacteroidetes_Flavobacteriia_Flavobacteriales_Schleiferiaceae | 0.00 | 0.00 | 0.00 | 0.00 | 0.00 | 0.00 | 0.02 | 0.02 | 0.00 | 0.01 | 0.00 | 0.00 | 0.00 | 0.00 | 0.00 | 0.00 | 0.00 | 0.00 | 0.00 | 0.00 | 0.00 | 0.00 |
| Bacteroidetes_Sphingobacteriia_Sphingobacteriales_NS11-12_marine_group | 0.00 | 0.00 | 0.00 | 0.00 | 0.00 | 0.00 | 0.73 | 0.77 | 0.04 | 0.03 | 0.00 | 0.00 | 0.26 | 0.10 | 0.25 | 0.21 | 0.00 | 0.00 | 0.00 | 0.01 | 0.01 | 0.00 |
| Bacteroidetes_Sphingobacteriia_Sphingobacteriales_Saprospiraceae | 0.13 | 0.08 | 0.03 | 0.02 | 0.06 | 0.05 | 0.05 | 0.05 | 0.11 | 0.12 | 0.01 | 0.03 | 0.35 | 0.43 | 0.10 | 0.08 | 0.04 | 0.04 | 0.02 | 0.05 | 0.01 | 0.02 |
| Bacteroidetes_Sphingobacteriia_Sphingobacteriales_WCHB1-69 | 0.00 | 0.00 | 0.00 | 0.00 | 0.00 | 0.00 | 0.00 | 0.00 | 0.00 | 0.00 | 0.00 | 0.00 | 0.00 | 0.00 | 0.01 | 0.00 | 0.00 | 0.00 | 0.00 | 0.00 | 0.00 | 0.00 |
| Candidate_division_SR1 | 0.00 | 0.00 | 0.00 | 0.00 | 0.00 | 0.00 | 0.00 | 0.00 | 0.00 | 0.00 | 0.00 | 0.00 | 0.00 | 0.00 | 0.00 | 0.00 | 0.00 | 0.00 | 0.00 | 0.00 | 0.00 | 0.00 |
| Chloroflexi_JG30-KF-CM66 | 0.00 | 0.00 | 0.00 | 0.00 | 0.00 | 0.00 | 0.00 | 0.00 | 0.00 | 0.00 | 0.00 | 0.00 | 0.00 | 0.00 | 0.00 | 0.00 | 0.00 | 0.00 | 0.00 | 0.00 | 0.00 | 0.00 |
| Chloroflexi_SAR202_clade | 0.25 | 0.44 | 0.16 | 0.23 | 0.54 | 0.39 | 0.18 | 0.15 | 0.07 | 0.04 | 0.39 | 0.25 | 0.03 | 0.01 | 0.07 | 0.06 | 0.16 | 0.27 | 0.36 | 0.29 | 0.03 | 0.03 |
| Cyanobacteria | 0.00 | 0.00 | 0.00 | 0.00 | 0.00 | 0.00 | 0.02 | 0.02 | 0.00 | 0.01 | 0.00 | 0.00 | 0.01 | 0.00 | 0.01 | 0.00 | 0.00 | 0.00 | 0.00 | 0.00 | 0.00 | 0.00 |
| Cyanobacteria | 0.01 | 0.03 | 0.01 | 0.03 | 0.55 | 0.40 | 0.16 | 0.25 | 0.04 | 0.07 | 0.04 | 0.06 | 0.03 | 0.03 | 0.02 | 0.02 | 0.11 | 0.11 | 0.05 | 0.06 | 0.03 | 0.03 |
| Cyanobacteria_Cyanobacteria_SubsectionI_FamilyI | 3.99 | 6.88 | 4.77 | 7.26 | 2.37 | 1.90 | 1.12 | 2.31 | 0.26 | 0.56 | 7.22 | 5.28 | 0.11 | 0.10 | 0.02 | 0.01 | 2.17 | 2.32 | 0.80 | 2.38 | 0.59 | 1.06 |
| Cyanobacteria_Cyanobacteria_SubsectionIII_FamilyI | 0.00 | 0.00 | 0.00 | 0.03 | 0.00 | 0.00 | 0.00 | 0.00 | 0.00 | 0.00 | 0.00 | 0.00 | 0.00 | 0.00 | 0.00 | 0.00 | 0.00 | 0.00 | 0.00 | 0.00 | 0.00 | 0.00 |
| Cyanobacteria_ML635J-21_ML635J-21_ML635J-21 | 0.00 | 0.00 | 0.00 | 0.00 | 0.00 | 0.00 | 0.00 | 0.00 | 0.00 | 0.00 | 0.00 | 0.00 | 0.00 | 0.00 | 0.00 | 0.01 | 0.00 | 0.00 | 0.00 | 0.00 | 0.00 | 0.00 |
| Firmicutes_Bacilli_Bacillales_Bacillaceae | 0.01 | 0.01 | 0.01 | 0.03 | 0.00 | 0.02 | 0.01 | 0.03 | 0.00 | 0.03 | 0.01 | 0.03 | 0.01 | 0.01 | 0.00 | 0.01 | 0.02 | 0.01 | 0.01 | 0.00 | 0.00 | 0.01 |
| Firmicutes_Bacilli_Bacillales_Staphylococcaceae | 0.05 | 0.00 | 0.04 | 0.00 | 0.00 | 0.04 | 0.00 | 0.00 | 0.01 | 0.00 | 0.01 | 0.00 | 0.00 | 0.00 | 0.00 | 0.00 | 0.00 | 0.00 | 0.00 | 0.00 | 0.01 | 0.00 |
| Firmicutes_Bacilli_Lactobacillales_Carnobacteriaceae | 0.00 | 0.00 | 0.00 | 0.00 | 0.00 | 0.00 | 0.00 | 0.00 | 0.01 | 0.00 | 0.00 | 0.00 | 0.00 | 0.01 | 0.00 | 0.00 | 0.00 | 0.00 | 0.00 | 0.00 | 0.00 | 0.00 |
| Firmicutes_Bacilli_Lactobacillales_Enterococcaceae | 0.01 | 0.00 | 0.00 | 0.02 | 0.01 | 0.01 | 0.02 | 0.00 | 0.00 | 0.01 | 0.00 | 0.01 | 0.00 | 0.00 | 0.00 | 0.01 | 0.00 | 0.01 | 0.00 | 0.01 | 0.00 | 0.01 |
| Firmicutes_Bacilli_Lactobacillales_Lactobacillaceae | 0.07 | 0.05 | 0.05 | 0.10 | 0.06 | 0.05 | 0.05 | 0.05 | 0.05 | 0.06 | 0.05 | 0.12 | 0.10 | 0.08 | 0.04 | 0.04 | 0.03 | 0.04 | 0.05 | 0.02 | 0.06 | 0.07 |
| Firmicutes_Bacilli_Lactobacillales_Streptococcaceae | 0.03 | 0.04 | 0.02 | 0.04 | 0.02 | 0.01 | 0.04 | 0.02 | 0.02 | 0.03 | 0.01 | 0.03 | 0.03 | 0.03 | 0.02 | 0.06 | 0.01 | 0.02 | 0.03 | 0.01 | 0.01 | 0.03 |
| Firmicutes_Clostridia_Clostridiales | 0.00 | 0.00 | 0.00 | 0.00 | 0.00 | 0.00 | 0.00 | 0.00 | 0.00 | 0.00 | 0.00 | 0.00 | 0.00 | 0.00 | 0.00 | 0.00 | 0.00 | 0.00 | 0.00 | 0.00 | 0.00 | 0.00 |
| Firmicutes_Clostridia_Clostridiales_Christensenellaceae | 0.00 | 0.01 | 0.00 | 0.00 | 0.01 | 0.02 | 0.01 | 0.00 | 0.00 | 0.01 | 0.01 | 0.00 | 0.01 | 0.01 | 0.01 | 0.01 | 0.00 | 0.01 | 0.01 | 0.00 | 0.01 | 0.01 |
| Firmicutes_Clostridia_Clostridiales_Clostridiaceae_1 | 0.06 | 0.06 | 0.07 | 0.09 | 0.06 | 0.07 | 0.07 | 0.05 | 0.06 | 0.06 | 0.05 | 0.09 | 0.06 | 0.09 | 0.03 | 0.08 | 0.06 | 0.06 | 0.06 | 0.02 | 0.04 | 0.04 |
| Firmicutes_Clostridia_Clostridiales_Lachnospiraceae | 0.04 | 0.05 | 0.02 | 0.11 | 0.04 | 0.05 | 0.03 | 0.04 | 0.02 | 0.05 | 0.03 | 0.05 | 0.04 | 0.08 | 0.02 | 0.03 | 0.04 | 0.03 | 0.04 | 0.03 | 0.04 | 0.05 |
| Firmicutes_Clostridia_Clostridiales_Ruminococcaceae | 0.03 | 0.02 | 0.03 | 0.06 | 0.03 | 0.01 | 0.03 | 0.02 | 0.02 | 0.02 | 0.02 | 0.05 | 0.03 | 0.05 | 0.02 | 0.05 | 0.03 | 0.03 | 0.03 | 0.02 | 0.02 | 0.03 |
| Firmicutes_Erysipelotrichia_Erysipelotrichales_Erysipelotrichaceae | 0.00 | 0.00 | 0.01 | 0.00 | 0.01 | 0.01 | 0.01 | 0.02 | 0.01 | 0.01 | 0.00 | 0.02 | 0.01 | 0.02 | 0.01 | 0.02 | 0.00 | 0.01 | 0.01 | 0.01 | 0.01 | 0.01 |
| Firmicutes_Negativicutes_Selenomonadales_Acidaminococcaceae | 0.00 | 0.00 | 0.00 | 0.00 | 0.01 | 0.00 | 0.00 | 0.00 | 0.00 | 0.00 | 0.01 | 0.00 | 0.00 | 0.00 | 0.00 | 0.00 | 0.00 | 0.00 | 0.00 | 0.01 | 0.00 | 0.00 |
| Firmicutes_Negativicutes_Selenomonadales_Veillonellaceae | 0.11 | 0.08 | 0.04 | 0.25 | 0.11 | 0.09 | 0.09 | 0.13 | 0.09 | 0.12 | 0.08 | 0.11 | 0.03 | 0.02 | 0.02 | 0.02 | 0.03 | 0.01 | 0.02 | 0.02 | 0.02 | 0.02 |
| Fusobacteria_Fusobacteriia_Fusobacteriales_Fusobacteriaceae | 0.00 | 0.00 | 0.00 | 0.00 | 0.00 | 0.00 | 0.00 | 0.01 | 0.01 | 0.00 | 0.00 | 0.00 | 0.00 | 0.00 | 0.00 | 0.00 | 0.00 | 0.00 | 0.00 | 0.00 | 0.01 | 0.02 |
| Gemmatimonadetes_Gemmatimonadetes_BD2-11_terrestrial_group | 0.00 | 0.00 | 0.00 | 0.00 | 0.02 | 0.03 | 0.01 | 0.02 | 0.00 | 0.00 | 0.02 | 0.04 | 0.00 | 0.00 | 0.02 | 0.01 | 0.01 | 0.01 | 0.00 | 0.01 | 0.01 | 0.00 |
| Gracilibacteria | 0.00 | 0.00 | 0.00 | 0.00 | 0.00 | 0.00 | 0.00 | 0.00 | 0.00 | 0.00 | 0.00 | 0.00 | 0.00 | 0.00 | 0.01 | 0.02 | 0.00 | 0.00 | 0.01 | 0.00 | 0.00 | 0.00 |
| Hydrogenedentes | 0.00 | 0.00 | 0.00 | 0.00 | 0.00 | 0.00 | 0.01 | 0.01 | 0.00 | 0.00 | 0.00 | 0.00 | 0.00 | 0.00 | 0.01 | 0.00 | 0.00 | 0.00 | 0.00 | 0.00 | 0.00 | 0.00 |
| Lentisphaerae_LD1-PB3 | 0.00 | 0.00 | 0.00 | 0.00 | 0.00 | 0.00 | 0.00 | 0.00 | 0.00 | 0.00 | 0.00 | 0.00 | 0.00 | 0.00 | 0.00 | 0.00 | 0.00 | 0.00 | 0.00 | 0.00 | 0.00 | 0.00 |
| Lentisphaerae_Lentisphaerae_Lentisphaerae_Lentisphaerae | 0.00 | 0.00 | 0.00 | 0.00 | 0.00 | 0.00 | 0.00 | 0.00 | 0.00 | 0.00 | 0.00 | 0.00 | 0.00 | 0.02 | 0.00 | 0.00 | 0.00 | 0.00 | 0.00 | 0.00 | 0.00 | 0.00 |
| Lentisphaerae_Lentisphaeria_Lentisphaerales_Lentisphaeraceae | 0.00 | 0.00 | 0.00 | 0.00 | 0.03 | 0.02 | 0.00 | 0.00 | 0.16 | 0.37 | 0.01 | 0.01 | 0.07 | 0.12 | 0.02 | 0.02 | 0.02 | 0.05 | 0.01 | 0.01 | 0.02 | 0.03 |
| Lentisphaerae_Oligosphaeria | 0.00 | 0.00 | 0.00 | 0.00 | 0.00 | 0.00 | 0.00 | 0.00 | 0.00 | 0.00 | 0.00 | 0.00 | 0.00 | 0.00 | 0.00 | 0.00 | 0.00 | 0.00 | 0.00 | 0.00 | 0.00 | 0.00 |
| Marinimicrobia_(SAR406_clade) | 7.75 | 8.75 | 6.17 | 6.08 | 6.18 | 5.85 | 5.28 | 3.98 | 2.31 | 2.66 | 5.49 | 6.57 | 2.47 | 3.20 | 0.88 | 0.96 | 4.40 | 6.38 | 8.35 | 7.03 | 1.90 | 2.38 |
| Parcubacteria | 0.00 | 0.00 | 0.00 | 0.00 | 0.00 | 0.01 | 0.00 | 0.00 | 0.00 | 0.00 | 0.00 | 0.00 | 0.00 | 0.00 | 0.00 | 0.00 | 0.00 | 0.00 | 0.00 | 0.00 | 0.00 | 0.00 |
| PAUC34f | 0.00 | 0.00 | 0.00 | 0.00 | 0.01 | 0.00 | 0.00 | 0.00 | 0.00 | 0.00 | 0.00 | 0.01 | 0.00 | 0.00 | 0.01 | 0.01 | 0.00 | 0.00 | 0.00 | 0.00 | 0.00 | 0.00 |
| Planctomycetes | 0.00 | 0.00 | 0.00 | 0.00 | 0.00 | 0.00 | 0.00 | 0.00 | 0.00 | 0.00 | 0.00 | 0.00 | 0.00 | 0.00 | 0.00 | 0.00 | 0.00 | 0.00 | 0.00 | 0.00 | 0.00 | 0.00 |
| Planctomycetes_028H05-P-BN-P5 | 0.00 | 0.00 | 0.00 | 0.00 | 0.00 | 0.00 | 0.00 | 0.00 | 0.00 | 0.00 | 0.00 | 0.00 | 0.00 | 0.00 | 0.00 | 0.00 | 0.00 | 0.00 | 0.00 | 0.00 | 0.00 | 0.00 |
| Planctomycetes_OM190 | 0.00 | 0.00 | 0.00 | 0.00 | 0.02 | 0.02 | 0.17 | 0.31 | 0.07 | 0.08 | 0.02 | 0.02 | 0.13 | 0.05 | 0.16 | 0.22 | 0.03 | 0.01 | 0.04 | 0.07 | 0.04 | 0.03 |
| Planctomycetes_Phycisphaerae_Phycisphaerales_Phycisphaeraceae | 0.17 | 0.20 | 0.28 | 0.37 | 0.17 | 0.26 | 0.58 | 0.73 | 0.27 | 0.30 | 0.35 | 0.36 | 0.37 | 0.19 | 0.47 | 0.37 | 0.57 | 0.45 | 0.89 | 1.51 | 0.34 | 0.31 |
| Planctomycetes_Pla3_lineage | 0.00 | 0.00 | 0.00 | 0.00 | 0.01 | 0.00 | 0.02 | 0.04 | 0.00 | 0.00 | 0.00 | 0.00 | 0.00 | 0.00 | 0.01 | 0.00 | 0.01 | 0.01 | 0.02 | 0.03 | 0.01 | 0.01 |
| Planctomycetes_Planctomycetacia_Brocadiales_Brocadiaceae | 0.00 | 0.00 | 0.00 | 0.00 | 0.00 | 0.00 | 0.00 | 0.00 | 0.00 | 0.00 | 0.00 | 0.00 | 0.00 | 0.00 | 0.00 | 0.00 | 0.00 | 0.00 | 0.00 | 0.00 | 0.01 | 0.01 |
| Planctomycetes_Planctomycetacia_Planctomycetales_Planctomycetaceae | 0.20 | 0.30 | 0.29 | 0.31 | 0.31 | 0.38 | 0.83 | 1.98 | 0.39 | 0.34 | 0.19 | 0.11 | 0.24 | 0.13 | 0.47 | 0.48 | 0.22 | 0.13 | 0.08 | 0.41 | 0.27 | 0.21 |
| Proteobacteria_AEGEAN-245 | 0.01 | 0.01 | 0.00 | 0.00 | 0.00 | 0.00 | 0.04 | 0.02 | 0.04 | 0.04 | 0.03 | 0.02 | 0.00 | 0.01 | 0.05 | 0.05 | 0.00 | 0.01 | 0.01 | 0.01 | 0.05 | 0.10 |
| Proteobacteria_Alphaproteobacteria | 0.03 | 0.05 | 0.07 | 0.04 | 0.14 | 0.14 | 0.10 | 0.17 | 0.01 | 0.03 | 0.11 | 0.12 | 0.06 | 0.05 | 0.06 | 0.07 | 0.13 | 0.08 | 0.08 | 0.11 | 0.06 | 0.09 |
| Proteobacteria_Alphaproteobacteria_Caulobacterales_Caulobacteraceae | 0.00 | 0.00 | 0.01 | 0.00 | 0.00 | 0.03 | 0.00 | 0.00 | 0.00 | 0.00 | 0.00 | 0.00 | 0.00 | 0.00 | 0.00 | 0.00 | 0.00 | 0.00 | 0.00 | 0.01 | 0.01 | 0.00 |
| Proteobacteria_Alphaproteobacteria_Caulobacterales_Hyphomonadaceae | 0.00 | 0.00 | 0.00 | 0.00 | 0.00 | 0.00 | 0.35 | 0.33 | 0.12 | 0.12 | 0.01 | 0.01 | 0.37 | 0.63 | 0.22 | 0.22 | 0.11 | 0.09 | 0.02 | 0.05 | 0.86 | 1.14 |
| Proteobacteria_Alphaproteobacteria_DB1-14_DB1-14 | 0.00 | 0.00 | 0.00 | 0.00 | 0.00 | 0.00 | 0.00 | 0.00 | 0.00 | 0.00 | 0.00 | 0.00 | 0.00 | 0.00 | 0.00 | 0.00 | 0.00 | 0.00 | 0.00 | 0.00 | 0.00 | 0.00 |
| Proteobacteria_Alphaproteobacteria_Kordiimonadales_Temperatibacteraceae | 0.00 | 0.00 | 0.00 | 0.00 | 0.00 | 0.02 | 0.00 | 0.00 | 0.00 | 0.00 | 0.00 | 0.00 | 0.00 | 0.00 | 0.00 | 0.00 | 0.00 | 0.00 | 0.00 | 0.00 | 0.00 | 0.00 |
| Proteobacteria_Alphaproteobacteria_OCS116_clade | 2.13 | 2.18 | 2.24 | 3.03 | 2.72 | 2.19 | 1.82 | 1.56 | 0.91 | 1.07 | 2.20 | 2.73 | 1.09 | 1.18 | 0.28 | 0.34 | 2.23 | 3.00 | 3.10 | 2.34 | 0.54 | 0.79 |
| Proteobacteria_Alphaproteobacteria_Parvularculales_Parvularculaceae | 0.00 | 0.00 | 0.00 | 0.01 | 0.00 | 0.00 | 0.00 | 0.00 | 0.00 | 0.00 | 0.00 | 0.00 | 0.00 | 0.00 | 0.00 | 0.00 | 0.00 | 0.01 | 0.00 | 0.00 | 0.00 | 0.00 |
| Proteobacteria_Alphaproteobacteria_Rhizobiales_Bradyrhizobiaceae | 0.00 | 0.00 | 0.00 | 0.01 | 0.00 | 0.00 | 0.00 | 0.00 | 0.00 | 0.00 | 0.00 | 0.00 | 0.00 | 0.00 | 0.00 | 0.00 | 0.00 | 0.00 | 0.00 | 0.00 | 0.00 | 0.00 |
| Proteobacteria_Alphaproteobacteria_Rhizobiales_Methylobacteriaceae | 0.00 | 0.00 | 0.00 | 0.00 | 0.00 | 0.00 | 0.00 | 0.00 | 0.00 | 0.00 | 0.00 | 0.00 | 0.00 | 0.00 | 0.00 | 0.00 | 0.00 | 0.00 | 0.00 | 0.00 | 0.00 | 0.00 |
| Proteobacteria_Alphaproteobacteria_Rhizobiales_Phyllobacteriaceae | 0.00 | 0.00 | 0.00 | 0.00 | 0.00 | 0.00 | 0.00 | 0.00 | 0.00 | 0.00 | 0.00 | 0.00 | 0.02 | 0.04 | 0.02 | 0.03 | 0.00 | 0.00 | 0.00 | 0.00 | 0.00 | 0.01 |
| Proteobacteria_Alphaproteobacteria_Rhizobiales_Rhizobiaceae | 0.00 | 0.00 | 0.00 | 0.00 | 0.00 | 0.00 | 0.01 | 0.00 | 0.00 | 0.00 | 0.00 | 0.00 | 0.00 | 0.00 | 0.00 | 0.00 | 0.01 | 0.00 | 0.01 | 0.02 | 0.03 | 0.01 |
| Proteobacteria_Alphaproteobacteria_Rhizobiales_Rhodobiaceae | 0.00 | 0.00 | 0.01 | 0.01 | 0.00 | 0.01 | 0.13 | 0.17 | 0.04 | 0.03 | 0.00 | 0.01 | 0.05 | 0.03 | 0.07 | 0.06 | 0.01 | 0.01 | 0.00 | 0.01 | 0.01 | 0.01 |
| Proteobacteria_Alphaproteobacteria_Rhodobacterales_Rhodobacteraceae | 7.55 | 5.77 | 7.71 | 8.82 | 5.43 | 7.33 | 14.66 | 15.55 | 14.04 | 11.19 | 6.48 | 5.63 | 10.74 | 9.14 | 16.50 | 20.20 | 7.80 | 6.79 | 5.62 | 7.27 | 20.18 | 20.73 |
| Proteobacteria_Alphaproteobacteria_Rhodospirillales | 0.02 | 0.00 | 0.01 | 0.01 | 0.04 | 0.04 | 0.11 | 0.15 | 0.01 | 0.04 | 0.07 | 0.07 | 0.01 | 0.01 | 0.03 | 0.05 | 0.06 | 0.02 | 0.06 | 0.07 | 0.06 | 0.06 |
| Proteobacteria_Alphaproteobacteria_Rhodospirillales_AT-s3-44 | 0.00 | 0.00 | 0.00 | 0.00 | 0.00 | 0.00 | 0.00 | 0.01 | 0.00 | 0.02 | 0.00 | 0.00 | 0.00 | 0.00 | 0.00 | 0.02 | 0.00 | 0.00 | 0.00 | 0.00 | 0.01 | 0.00 |
| Proteobacteria_Alphaproteobacteria_Rhodospirillales_Rhodospirillaceae | 5.08 | 4.44 | 6.48 | 3.86 | 2.81 | 3.65 | 2.61 | 1.98 | 1.03 | 1.03 | 3.88 | 3.46 | 0.96 | 1.02 | 0.46 | 0.48 | 3.46 | 3.17 | 3.51 | 4.04 | 0.93 | 0.93 |
| Proteobacteria_Alphaproteobacteria_Rickettsiales | 0.00 | 0.00 | 0.00 | 0.00 | 0.00 | 0.00 | 0.00 | 0.00 | 0.00 | 0.00 | 0.00 | 0.00 | 0.00 | 0.00 | 0.00 | 0.00 | 0.00 | 0.00 | 0.00 | 0.00 | 0.00 | 0.00 |
| Proteobacteria_Alphaproteobacteria_Rickettsiales | 0.00 | 0.01 | 0.00 | 0.01 | 0.03 | 0.01 | 0.00 | 0.00 | 0.01 | 0.00 | 0.00 | 0.01 | 0.00 | 0.00 | 0.00 | 0.02 | 0.01 | 0.03 | 0.01 | 0.01 | 0.00 | 0.01 |
| Proteobacteria_Alphaproteobacteria_Rickettsiales_EF100-94H03 | 0.00 | 0.00 | 0.00 | 0.00 | 0.00 | 0.00 | 0.00 | 0.01 | 0.01 | 0.00 | 0.01 | 0.01 | 0.00 | 0.00 | 0.01 | 0.00 | 0.00 | 0.00 | 0.00 | 0.00 | 0.00 | 0.00 |
| Proteobacteria_Alphaproteobacteria_Rickettsiales_Holosporaceae | 0.01 | 0.00 | 0.00 | 0.00 | 0.00 | 0.00 | 0.00 | 0.00 | 0.00 | 0.00 | 0.00 | 0.00 | 0.00 | 0.00 | 0.00 | 0.00 | 0.00 | 0.00 | 0.00 | 0.02 | 0.00 | 0.00 |
| Proteobacteria_Alphaproteobacteria_Rickettsiales_LR_A2-29 | 0.00 | 0.00 | 0.00 | 0.00 | 0.00 | 0.00 | 0.00 | 0.00 | 0.00 | 0.00 | 0.00 | 0.00 | 0.00 | 0.00 | 0.00 | 0.00 | 0.00 | 0.00 | 0.00 | 0.00 | 0.00 | 0.00 |
| Proteobacteria_Alphaproteobacteria_Rickettsiales_LWSR-14 | 0.00 | 0.00 | 0.00 | 0.00 | 0.02 | 0.01 | 0.00 | 0.00 | 0.00 | 0.00 | 0.01 | 0.03 | 0.01 | 0.01 | 0.00 | 0.00 | 0.00 | 0.00 | 0.01 | 0.03 | 0.00 | 0.00 |
| Proteobacteria_Alphaproteobacteria_Rickettsiales_Rickettsiaceae | 0.01 | 0.03 | 0.01 | 0.03 | 0.07 | 0.01 | 0.00 | 0.00 | 0.00 | 0.03 | 0.02 | 0.02 | 0.02 | 0.01 | 0.01 | 0.02 | 0.09 | 0.07 | 0.18 | 0.17 | 0.04 | 0.18 |
| Proteobacteria_Alphaproteobacteria_Rickettsiales_Rickettsiales_Incertae_Sedis | 0.01 | 0.06 | 0.01 | 0.01 | 0.03 | 0.02 | 0.00 | 0.00 | 0.01 | 0.01 | 0.03 | 0.07 | 0.02 | 0.00 | 0.00 | 0.00 | 0.02 | 0.07 | 0.03 | 0.07 | 0.02 | 0.02 |
| Proteobacteria_Alphaproteobacteria_Rickettsiales_S25-593 | 1.04 | 0.91 | 0.88 | 0.56 | 0.94 | 1.26 | 0.06 | 0.08 | 0.06 | 0.10 | 0.40 | 0.37 | 0.05 | 0.05 | 0.03 | 0.01 | 0.23 | 0.22 | 0.19 | 0.25 | 0.05 | 0.03 |
| Proteobacteria_Alphaproteobacteria_Rickettsiales_SAR116_clade | 7.96 | 6.76 | 8.55 | 6.92 | 5.38 | 6.29 | 2.43 | 1.96 | 4.81 | 4.37 | 3.00 | 3.22 | 2.70 | 2.47 | 0.97 | 1.10 | 4.67 | 3.33 | 2.79 | 2.98 | 1.15 | 1.29 |
| Proteobacteria_Alphaproteobacteria_Rickettsiales_SHWN-night2 | 0.00 | 0.00 | 0.00 | 0.00 | 0.02 | 0.02 | 0.18 | 0.33 | 0.01 | 0.01 | 0.03 | 0.05 | 0.01 | 0.00 | 0.01 | 0.00 | 0.04 | 0.01 | 0.02 | 0.02 | 0.01 | 0.01 |
| Proteobacteria_Alphaproteobacteria_SAR11_clade | 0.31 | 1.49 | 0.53 | 0.64 | 1.29 | 0.95 | 0.22 | 0.21 | 0.24 | 0.36 | 0.70 | 0.62 | 0.12 | 0.23 | 0.06 | 0.10 | 0.76 | 1.15 | 1.55 | 1.52 | 0.12 | 0.20 |
| Proteobacteria_Alphaproteobacteria_SAR11_clade_Deep_1 | 0.01 | 0.00 | 0.01 | 0.01 | 0.01 | 0.00 | 0.15 | 0.19 | 0.09 | 0.18 | 0.06 | 0.08 | 0.04 | 0.04 | 0.07 | 0.09 | 0.03 | 0.05 | 0.01 | 0.01 | 0.02 | 0.08 |
| Proteobacteria_Alphaproteobacteria_SAR11_clade_Surface_1 | 1.94 | 6.36 | 4.38 | 3.41 | 8.09 | 6.05 | 6.59 | 5.79 | 6.40 | 9.40 | 7.58 | 6.88 | 5.21 | 7.83 | 0.64 | 0.75 | 6.39 | 9.59 | 9.66 | 9.69 | 1.10 | 2.57 |
| Proteobacteria_Alphaproteobacteria_SAR11_clade_Surface_4 | 0.38 | 0.34 | 0.47 | 0.38 | 0.48 | 0.58 | 0.08 | 0.07 | 0.09 | 0.15 | 0.59 | 0.55 | 0.06 | 0.09 | 0.04 | 0.06 | 0.69 | 0.63 | 0.87 | 0.72 | 0.12 | 0.13 |
| Proteobacteria_Alphaproteobacteria_SB1-18 | 0.00 | 0.00 | 0.00 | 0.00 | 0.00 | 0.00 | 0.34 | 0.40 | 0.59 | 0.91 | 0.04 | 0.04 | 0.32 | 0.52 | 0.21 | 0.21 | 0.00 | 0.00 | 0.00 | 0.00 | 0.00 | 0.00 |
| Proteobacteria_Alphaproteobacteria_Sphingomonadales | 0.00 | 0.00 | 0.01 | 0.01 | 0.00 | 0.00 | 0.00 | 0.00 | 0.00 | 0.00 | 0.00 | 0.01 | 0.00 | 0.00 | 0.00 | 0.00 | 0.00 | 0.00 | 0.00 | 0.00 | 0.00 | 0.00 |
| Proteobacteria_Alphaproteobacteria_Sphingomonadales_Erythrobacteraceae | 0.04 | 0.08 | 0.02 | 0.04 | 0.02 | 0.01 | 0.00 | 0.00 | 0.00 | 0.00 | 0.00 | 0.00 | 0.00 | 0.01 | 0.00 | 0.00 | 0.00 | 0.01 | 0.00 | 0.01 | 0.00 | 0.00 |
| Proteobacteria_Alphaproteobacteria_Sphingomonadales_Sphingomonadaceae | 1.34 | 1.00 | 0.00 | 0.00 | 0.88 | 1.31 | 0.23 | 0.39 | 0.65 | 0.74 | 0.33 | 0.38 | 0.33 | 0.25 | 0.00 | 0.00 | 0.00 | 0.00 | 0.01 | 0.00 | 0.01 | 0.00 |
| Proteobacteria_Betaproteobacteria | 0.04 | 0.06 | 0.05 | 0.05 | 0.06 | 0.10 | 0.70 | 0.61 | 0.21 | 0.21 | 0.05 | 0.05 | 0.18 | 0.24 | 0.18 | 0.15 | 0.09 | 0.07 | 0.02 | 0.01 | 0.06 | 0.06 |
| Proteobacteria_Betaproteobacteria_Burkholderiales | 0.00 | 0.00 | 0.00 | 0.00 | 0.00 | 0.00 | 0.00 | 0.00 | 0.00 | 0.00 | 0.00 | 0.00 | 0.00 | 0.00 | 0.00 | 0.00 | 0.00 | 0.00 | 0.00 | 0.01 | 0.00 | 0.00 |
| Proteobacteria_Betaproteobacteria_Burkholderiales_Alcaligenaceae | 0.54 | 0.41 | 0.45 | 0.33 | 0.05 | 0.08 | 0.02 | 0.00 | 0.01 | 0.00 | 0.11 | 0.02 | 0.01 | 0.00 | 0.00 | 0.00 | 0.06 | 0.02 | 0.02 | 0.03 | 0.01 | 0.00 |
| Proteobacteria_Betaproteobacteria_Burkholderiales_Burkholderiaceae | 0.00 | 0.00 | 0.00 | 0.00 | 0.00 | 0.00 | 0.00 | 0.02 | 0.00 | 0.00 | 0.00 | 0.00 | 0.00 | 0.00 | 0.00 | 0.00 | 0.00 | 0.00 | 0.00 | 0.00 | 0.00 | 0.00 |
| Proteobacteria_Betaproteobacteria_Burkholderiales_Comamonadaceae | 0.01 | 0.01 | 0.04 | 0.01 | 0.01 | 0.01 | 0.02 | 0.02 | 0.06 | 0.04 | 0.01 | 0.01 | 0.00 | 0.00 | 0.00 | 0.00 | 0.01 | 0.01 | 0.01 | 0.01 | 0.00 | 0.01 |
| Proteobacteria_Betaproteobacteria_Methylophilales_Methylophilaceae | 0.00 | 0.00 | 0.00 | 0.01 | 0.08 | 0.12 | 1.19 | 0.97 | 0.54 | 0.73 | 0.11 | 0.16 | 0.72 | 0.69 | 0.20 | 0.19 | 0.61 | 0.80 | 0.92 | 0.80 | 0.26 | 0.26 |
| Proteobacteria_Betaproteobacteria_Nitrosomonadales_Nitrosomonadaceae | 0.00 | 0.00 | 0.00 | 0.00 | 0.01 | 0.00 | 0.01 | 0.01 | 0.00 | 0.00 | 0.01 | 0.01 | 0.00 | 0.00 | 0.01 | 0.00 | 0.00 | 0.01 | 0.00 | 0.00 | 0.00 | 0.01 |
| Proteobacteria_Deltaproteobacteria | 0.00 | 0.00 | 0.00 | 0.00 | 0.00 | 0.00 | 0.00 | 0.00 | 0.00 | 0.00 | 0.00 | 0.00 | 0.00 | 0.00 | 0.00 | 0.00 | 0.00 | 0.00 | 0.00 | 0.00 | 0.00 | 0.00 |
| Proteobacteria_Deltaproteobacteria | 0.01 | 0.02 | 0.05 | 0.07 | 0.01 | 0.01 | 0.13 | 0.25 | 0.01 | 0.00 | 0.03 | 0.02 | 0.00 | 0.00 | 0.01 | 0.00 | 0.00 | 0.01 | 0.01 | 0.00 | 0.02 | 0.01 |
| Proteobacteria_Deltaproteobacteria_Bdellovibrionales_Bacteriovoracaceae | 0.04 | 0.03 | 0.07 | 0.06 | 0.01 | 0.03 | 0.10 | 0.09 | 0.03 | 0.04 | 0.02 | 0.02 | 0.01 | 0.02 | 0.01 | 0.06 | 0.02 | 0.01 | 0.01 | 0.04 | 0.00 | 0.01 |
| Proteobacteria_Deltaproteobacteria_Bdellovibrionales_Bdellovibrionaceae | 0.48 | 0.58 | 0.58 | 0.64 | 0.08 | 0.12 | 0.48 | 0.68 | 0.14 | 0.22 | 0.44 | 0.52 | 0.32 | 0.27 | 0.31 | 0.33 | 0.14 | 0.15 | 0.25 | 0.51 | 0.12 | 0.11 |
| Proteobacteria_Deltaproteobacteria_Desulfarculales_Desulfarculaceae | 0.00 | 0.00 | 0.00 | 0.00 | 0.00 | 0.00 | 0.00 | 0.00 | 0.00 | 0.00 | 0.00 | 0.00 | 0.00 | 0.00 | 0.00 | 0.00 | 0.00 | 0.00 | 0.00 | 0.00 | 0.00 | 0.00 |
| Proteobacteria_Deltaproteobacteria_Desulfobacterales_Nitrospinaceae | 0.00 | 0.00 | 0.00 | 0.00 | 0.00 | 0.00 | 0.02 | 0.02 | 0.00 | 0.01 | 0.02 | 0.01 | 0.00 | 0.01 | 0.07 | 0.06 | 0.00 | 0.00 | 0.01 | 0.01 | 0.05 | 0.02 |
| Proteobacteria_Deltaproteobacteria_Desulfovibrionales_Desulfovibrionaceae | 0.00 | 0.00 | 0.00 | 0.00 | 0.00 | 0.00 | 0.00 | 0.00 | 0.00 | 0.01 | 0.00 | 0.00 | 0.00 | 0.01 | 0.00 | 0.00 | 0.00 | 0.00 | 0.00 | 0.00 | 0.00 | 0.00 |
| Proteobacteria_Deltaproteobacteria_Desulfuromonadales | 0.00 | 0.00 | 0.00 | 0.00 | 0.00 | 0.00 | 0.00 | 0.00 | 0.00 | 0.00 | 0.00 | 0.00 | 0.00 | 0.00 | 0.00 | 0.00 | 0.00 | 0.00 | 0.00 | 0.00 | 0.00 | 0.00 |
| Proteobacteria_Deltaproteobacteria_Desulfuromonadales | 0.01 | 0.00 | 0.01 | 0.01 | 0.00 | 0.00 | 0.01 | 0.00 | 0.00 | 0.00 | 0.00 | 0.00 | 0.00 | 0.00 | 0.00 | 0.00 | 0.00 | 0.00 | 0.00 | 0.00 | 0.00 | 0.00 |
| Proteobacteria_Deltaproteobacteria_Desulfuromonadales_GR-WP33-58 | 0.07 | 0.08 | 0.43 | 0.59 | 0.02 | 0.03 | 0.18 | 0.27 | 0.04 | 0.07 | 0.27 | 0.32 | 0.27 | 0.11 | 0.22 | 0.20 | 0.04 | 0.03 | 0.04 | 0.05 | 0.07 | 0.05 |
| Proteobacteria_Deltaproteobacteria_Myxococcales_BIrii41 | 0.01 | 0.05 | 0.01 | 0.04 | 0.01 | 0.01 | 0.00 | 0.00 | 0.00 | 0.00 | 0.01 | 0.01 | 0.01 | 0.01 | 0.01 | 0.00 | 0.01 | 0.01 | 0.00 | 0.01 | 0.01 | 0.00 |
| Proteobacteria_Deltaproteobacteria_Myxococcales_Blfdi19 | 0.01 | 0.01 | 0.14 | 0.07 | 0.04 | 0.03 | 0.13 | 0.21 | 0.01 | 0.00 | 0.03 | 0.07 | 0.01 | 0.03 | 0.00 | 0.01 | 0.03 | 0.04 | 0.06 | 0.10 | 0.02 | 0.01 |
| Proteobacteria_Deltaproteobacteria_Myxococcales_Eel-36e1D6 | 0.00 | 0.00 | 0.00 | 0.00 | 0.00 | 0.00 | 0.00 | 0.00 | 0.00 | 0.00 | 0.00 | 0.00 | 0.00 | 0.00 | 0.00 | 0.01 | 0.00 | 0.00 | 0.00 | 0.00 | 0.00 | 0.00 |
| Proteobacteria_Deltaproteobacteria_Myxococcales_Myxococcales | 0.00 | 0.00 | 0.01 | 0.01 | 0.03 | 0.01 | 0.03 | 0.08 | 0.00 | 0.00 | 0.00 | 0.00 | 0.00 | 0.00 | 0.00 | 0.00 | 0.00 | 0.00 | 0.00 | 0.01 | 0.00 | 0.00 |
| Proteobacteria_Deltaproteobacteria_Myxococcales_Nannocystaceae | 0.00 | 0.00 | 0.00 | 0.00 | 0.00 | 0.00 | 0.00 | 0.00 | 0.00 | 0.00 | 0.00 | 0.00 | 0.00 | 0.00 | 0.00 | 0.00 | 0.00 | 0.00 | 0.00 | 0.00 | 0.00 | 0.00 |
| Proteobacteria_Deltaproteobacteria_Myxococcales_P3OB-42 | 0.06 | 0.03 | 0.15 | 0.17 | 0.04 | 0.06 | 0.11 | 0.12 | 0.03 | 0.01 | 0.03 | 0.06 | 0.01 | 0.01 | 0.02 | 0.01 | 0.03 | 0.00 | 0.06 | 0.09 | 0.02 | 0.01 |
| Proteobacteria_Deltaproteobacteria_Myxococcales_Sandaracinaceae | 0.00 | 0.00 | 0.00 | 0.00 | 0.00 | 0.00 | 0.00 | 0.00 | 0.00 | 0.00 | 0.00 | 0.00 | 0.00 | 0.00 | 0.00 | 0.00 | 0.00 | 0.00 | 0.00 | 0.00 | 0.00 | 0.00 |
| Proteobacteria_Deltaproteobacteria_Oligoflexales_Oligoflexaceae | 0.08 | 0.11 | 0.13 | 0.14 | 0.08 | 0.07 | 0.02 | 0.02 | 0.03 | 0.05 | 0.05 | 0.05 | 0.01 | 0.01 | 0.01 | 0.02 | 0.10 | 0.13 | 0.02 | 0.08 | 0.02 | 0.01 |
| Proteobacteria_Deltaproteobacteria_SAR324_clade(Marine_group_B) | 0.51 | 0.58 | 0.29 | 0.54 | 0.47 | 1.10 | 0.57 | 0.30 | 0.46 | 0.37 | 0.95 | 0.64 | 0.13 | 0.07 | 0.55 | 0.52 | 0.24 | 0.20 | 0.29 | 0.17 | 0.30 | 0.25 |
| Proteobacteria_Deltaproteobacteria_Sh765B-TzT-29 | 0.00 | 0.00 | 0.00 | 0.00 | 0.01 | 0.01 | 0.01 | 0.02 | 0.01 | 0.00 | 0.01 | 0.01 | 0.01 | 0.00 | 0.06 | 0.05 | 0.01 | 0.01 | 0.02 | 0.01 | 0.05 | 0.05 |
| Proteobacteria_Epsilonproteobacteria_Campylobacterales_Campylobacteraceae | 0.01 | 0.00 | 0.00 | 0.03 | 0.00 | 0.00 | 0.00 | 0.01 | 0.01 | 0.01 | 0.02 | 0.05 | 0.01 | 0.01 | 0.01 | 0.00 | 0.01 | 0.01 | 0.01 | 0.02 | 0.04 | 0.03 |
| Proteobacteria_Gammaproteobacteria_Aeromonadales_Succinivibrionaceae | 0.00 | 0.00 | 0.00 | 0.01 | 0.00 | 0.00 | 0.00 | 0.00 | 0.00 | 0.00 | 0.01 | 0.00 | 0.00 | 0.01 | 0.00 | 0.00 | 0.00 | 0.00 | 0.00 | 0.00 | 0.00 | 0.00 |
| Proteobacteria_Gammaproteobacteria_Alteromonadales | 0.06 | 0.11 | 0.07 | 0.12 | 0.07 | 0.10 | 0.00 | 0.00 | 0.04 | 0.05 | 0.00 | 0.01 | 0.11 | 0.16 | 0.00 | 0.02 | 0.02 | 0.03 | 0.02 | 0.02 | 0.00 | 0.00 |
| Proteobacteria_Gammaproteobacteria_Alteromonadales_Alteromonadaceae | 0.12 | 0.13 | 0.06 | 0.05 | 0.04 | 0.02 | 0.00 | 0.00 | 0.09 | 0.06 | 0.04 | 0.02 | 0.12 | 0.10 | 0.54 | 0.07 | 0.35 | 0.07 | 0.30 | 0.09 | 0.71 | 0.01 |
| Proteobacteria_Gammaproteobacteria_Alteromonadales_Celerinatantimonadaceae | 0.00 | 0.00 | 0.00 | 0.01 | 0.00 | 0.00 | 0.00 | 0.00 | 0.00 | 0.00 | 0.00 | 0.01 | 0.00 | 0.00 | 0.00 | 0.00 | 0.00 | 0.00 | 0.00 | 0.01 | 0.00 | 0.00 |
| Proteobacteria_Gammaproteobacteria_Alteromonadales_Colwelliaceae | 0.04 | 0.04 | 0.01 | 0.02 | 0.04 | 0.01 | 0.04 | 0.03 | 0.01 | 0.05 | 0.01 | 0.01 | 0.07 | 0.12 | 0.06 | 0.16 | 0.03 | 0.09 | 0.01 | 0.09 | 0.02 | 0.04 |
| Proteobacteria_Gammaproteobacteria_Alteromonadales_Idiomarinaceae | 0.00 | 0.00 | 0.00 | 0.00 | 0.00 | 0.07 | 0.03 | 0.00 | 0.15 | 0.01 | 0.03 | 0.00 | 0.04 | 0.01 | 0.05 | 0.01 | 0.10 | 0.06 | 0.04 | 0.09 | 0.03 | 0.01 |
| Proteobacteria_Gammaproteobacteria_Alteromonadales_Moritellaceae | 0.00 | 0.00 | 0.00 | 0.00 | 0.00 | 0.00 | 0.00 | 0.00 | 0.00 | 0.00 | 0.00 | 0.00 | 0.00 | 0.00 | 0.01 | 0.00 | 0.00 | 0.00 | 0.00 | 0.00 | 0.00 | 0.00 |
| Proteobacteria_Gammaproteobacteria_Alteromonadales_Pseudoalteromonadaceae | 0.13 | 0.11 | 0.10 | 0.19 | 0.14 | 0.07 | 0.08 | 0.03 | 0.32 | 0.02 | 0.03 | 0.02 | 0.10 | 0.04 | 0.45 | 0.03 | 0.17 | 0.07 | 0.05 | 0.09 | 0.10 | 0.06 |
| Proteobacteria_Gammaproteobacteria_Alteromonadales_Psychromonadaceae | 0.00 | 0.00 | 0.00 | 0.00 | 0.00 | 0.00 | 0.00 | 0.00 | 0.00 | 0.00 | 0.00 | 0.00 | 0.00 | 0.00 | 0.00 | 0.00 | 0.00 | 0.00 | 0.00 | 0.01 | 0.03 | 0.04 |
| Proteobacteria_Gammaproteobacteria_Alteromonadales_Shewanellaceae | 0.00 | 0.00 | 0.01 | 0.04 | 0.01 | 0.02 | 0.00 | 0.00 | 0.01 | 0.00 | 0.01 | 0.00 | 0.01 | 0.00 | 0.00 | 0.00 | 0.00 | 0.00 | 0.01 | 0.00 | 0.02 | 0.01 |
| Proteobacteria_Gammaproteobacteria_Arenicellales_Arenicellaceae | 0.00 | 0.00 | 0.00 | 0.01 | 0.00 | 0.00 | 0.00 | 0.00 | 0.00 | 0.00 | 0.00 | 0.00 | 0.03 | 0.06 | 0.02 | 0.04 | 0.00 | 0.00 | 0.00 | 0.00 | 0.01 | 0.00 |
| Proteobacteria_Gammaproteobacteria_BD7-8_marine_group_BD7-8_marine_group | 0.00 | 0.00 | 0.00 | 0.00 | 0.00 | 0.00 | 0.00 | 0.00 | 0.00 | 0.00 | 0.00 | 0.00 | 0.00 | 0.00 | 0.01 | 0.01 | 0.00 | 0.00 | 0.00 | 0.00 | 0.00 | 0.00 |
| Proteobacteria_Gammaproteobacteria_Cellvibrionales | 0.00 | 0.01 | 0.01 | 0.02 | 0.01 | 0.00 | 0.00 | 0.00 | 0.00 | 0.00 | 0.01 | 0.01 | 0.00 | 0.00 | 0.00 | 0.00 | 0.00 | 0.01 | 0.00 | 0.00 | 0.00 | 0.00 |
| Proteobacteria_Gammaproteobacteria_Cellvibrionales_BD2-7 | 0.00 | 0.00 | 0.00 | 0.00 | 0.00 | 0.00 | 0.00 | 0.00 | 0.01 | 0.00 | 0.00 | 0.00 | 0.00 | 0.02 | 0.08 | 0.12 | 0.00 | 0.00 | 0.00 | 0.00 | 0.05 | 0.06 |
| Proteobacteria_Gammaproteobacteria_Cellvibrionales_Cellvibrionaceae | 0.02 | 0.00 | 0.00 | 0.01 | 0.03 | 0.00 | 0.02 | 0.00 | 0.00 | 0.00 | 0.00 | 0.00 | 0.07 | 0.12 | 0.01 | 0.02 | 0.00 | 0.01 | 0.00 | 0.00 | 0.00 | 0.01 |
| Proteobacteria_Gammaproteobacteria_Cellvibrionales_Halieaceae | 1.56 | 0.95 | 1.75 | 1.55 | 0.85 | 1.38 | 2.98 | 4.96 | 2.88 | 2.85 | 1.13 | 0.90 | 3.06 | 1.76 | 2.63 | 2.56 | 1.69 | 0.95 | 0.43 | 0.87 | 1.19 | 1.31 |
| Proteobacteria_Gammaproteobacteria_Cellvibrionales_Porticoccaceae | 0.04 | 0.09 | 0.35 | 0.40 | 0.00 | 0.00 | 0.37 | 0.37 | 0.59 | 0.56 | 0.04 | 0.07 | 3.47 | 3.22 | 3.01 | 3.53 | 0.53 | 0.42 | 0.42 | 0.43 | 0.84 | 0.74 |
| Proteobacteria_Gammaproteobacteria_Cellvibrionales_Spongiibacteraceae | 0.04 | 0.06 | 0.08 | 0.11 | 0.07 | 0.10 | 0.01 | 0.01 | 0.16 | 0.12 | 0.04 | 0.09 | 0.23 | 0.09 | 0.11 | 0.12 | 0.11 | 0.08 | 0.08 | 0.12 | 0.03 | 0.04 |
| Proteobacteria_Gammaproteobacteria_Chromatiales_Chromatiaceae | 0.01 | 0.00 | 0.00 | 0.00 | 0.02 | 0.00 | 0.00 | 0.00 | 0.00 | 0.00 | 0.00 | 0.00 | 0.01 | 0.00 | 0.00 | 0.00 | 0.00 | 0.00 | 0.00 | 0.00 | 0.00 | 0.00 |
| Proteobacteria_Gammaproteobacteria_Chromatiales_Ectothiorhodospiraceae | 0.00 | 0.00 | 0.00 | 0.00 | 0.00 | 0.00 | 0.00 | 0.00 | 0.00 | 0.00 | 0.00 | 0.00 | 0.00 | 0.00 | 0.00 | 0.00 | 0.00 | 0.00 | 0.00 | 0.00 | 0.00 | 0.01 |
| Proteobacteria_Gammaproteobacteria_Chromatiales_Granulosicoccaceae | 0.00 | 0.00 | 0.00 | 0.00 | 0.00 | 0.00 | 0.00 | 0.00 | 0.00 | 0.00 | 0.00 | 0.00 | 0.00 | 0.00 | 0.00 | 0.00 | 0.00 | 0.00 | 0.00 | 0.00 | 0.01 | 0.02 |
| Proteobacteria_Gammaproteobacteria_E01-9C-26_marine_group_E01-9C-26_marine_group | 0.00 | 0.00 | 0.00 | 0.00 | 0.09 | 0.17 | 0.75 | 1.46 | 0.21 | 0.18 | 0.13 | 0.13 | 0.15 | 0.09 | 0.40 | 0.29 | 0.26 | 0.11 | 0.09 | 0.16 | 0.26 | 0.23 |
| Proteobacteria_Gammaproteobacteria_Enterobacteriales_Enterobacteriaceae | 0.01 | 0.05 | 0.01 | 0.04 | 0.00 | 0.01 | 0.00 | 0.00 | 0.00 | 0.01 | 0.01 | 0.04 | 0.03 | 0.08 | 0.02 | 0.05 | 0.05 | 0.07 | 0.05 | 0.03 | 0.02 | 0.03 |
| Proteobacteria_Gammaproteobacteria_KI89A_clade | 0.32 | 0.21 | 0.34 | 0.51 | 0.31 | 0.48 | 0.34 | 0.30 | 0.45 | 0.43 | 0.32 | 0.34 | 0.72 | 0.55 | 0.35 | 0.39 | 0.43 | 0.23 | 0.22 | 0.28 | 0.18 | 0.14 |
| Proteobacteria_Gammaproteobacteria_Legionellales_Coxiellaceae | 0.02 | 0.03 | 0.06 | 0.09 | 0.01 | 0.02 | 0.08 | 0.21 | 0.03 | 0.02 | 0.09 | 0.13 | 0.01 | 0.02 | 0.03 | 0.05 | 0.01 | 0.01 | 0.02 | 0.00 | 0.03 | 0.01 |
| Proteobacteria_Gammaproteobacteria_Legionellales_Legionellaceae | 0.00 | 0.00 | 0.00 | 0.00 | 0.00 | 0.00 | 0.00 | 0.00 | 0.00 | 0.00 | 0.00 | 0.00 | 0.00 | 0.00 | 0.00 | 0.00 | 0.00 | 0.00 | 0.00 | 0.00 | 0.00 | 0.00 |
| Proteobacteria_Gammaproteobacteria_Methylococcales_Milano-WF1B-03 | 0.00 | 0.00 | 0.00 | 0.00 | 0.00 | 0.00 | 0.00 | 0.00 | 0.00 | 0.00 | 0.00 | 0.00 | 0.00 | 0.00 | 0.00 | 0.00 | 0.00 | 0.00 | 0.00 | 0.00 | 0.00 | 0.00 |
| Proteobacteria_Gammaproteobacteria_NKB5 | 0.00 | 0.00 | 0.00 | 0.00 | 0.01 | 0.00 | 0.01 | 0.00 | 0.00 | 0.00 | 0.00 | 0.00 | 0.00 | 0.00 | 0.00 | 0.00 | 0.00 | 0.00 | 0.00 | 0.00 | 0.00 | 0.00 |
| Proteobacteria_Gammaproteobacteria_Oceanospirillales | 2.22 | 2.96 | 1.90 | 2.27 | 5.78 | 4.58 | 0.02 | 0.03 | 0.07 | 0.09 | 1.65 | 2.14 | 0.01 | 0.03 | 0.01 | 0.02 | 0.61 | 1.50 | 0.95 | 0.99 | 0.05 | 0.05 |
| Proteobacteria_Gammaproteobacteria_Oceanospirillales | 0.04 | 0.03 | 0.03 | 0.07 | 4.13 | 3.84 | 9.84 | 9.12 | 4.76 | 5.93 | 4.96 | 7.27 | 6.24 | 6.76 | 7.64 | 9.02 | 6.31 | 6.19 | 6.22 | 4.68 | 8.21 | 9.11 |
| Proteobacteria_Gammaproteobacteria_Oceanospirillales_Alcanivoracaceae | 0.02 | 0.00 | 0.00 | 0.00 | 0.00 | 0.01 | 0.01 | 0.00 | 0.00 | 0.00 | 0.00 | 0.00 | 0.00 | 0.00 | 0.01 | 0.00 | 0.02 | 0.01 | 0.01 | 0.01 | 0.01 | 0.00 |
| Proteobacteria_Gammaproteobacteria_Oceanospirillales_Hahellaceae | 0.00 | 0.00 | 0.00 | 0.00 | 0.00 | 0.00 | 0.00 | 0.00 | 0.00 | 0.00 | 0.00 | 0.00 | 0.00 | 0.00 | 0.00 | 0.00 | 0.00 | 0.00 | 0.01 | 0.01 | 0.00 | 0.00 |
| Proteobacteria_Gammaproteobacteria_Oceanospirillales_Halomonadaceae | 0.00 | 0.00 | 0.01 | 0.00 | 0.31 | 0.76 | 0.17 | 0.02 | 0.31 | 0.09 | 0.09 | 0.04 | 0.07 | 0.02 | 0.25 | 0.12 | 0.46 | 0.37 | 0.15 | 0.53 | 0.21 | 0.06 |
| Proteobacteria_Gammaproteobacteria_Oceanospirillales_JL-ETNP-Y6 | 0.00 | 0.00 | 0.00 | 0.00 | 0.00 | 0.00 | 0.00 | 0.00 | 0.01 | 0.00 | 0.00 | 0.01 | 0.01 | 0.00 | 0.00 | 0.01 | 0.00 | 0.00 | 0.00 | 0.01 | 0.01 | 0.00 |
| Proteobacteria_Gammaproteobacteria_Oceanospirillales_Litoricolaceae | 0.01 | 0.00 | 0.03 | 0.01 | 0.00 | 0.00 | 0.06 | 0.07 | 0.00 | 0.02 | 0.00 | 0.00 | 0.07 | 0.03 | 0.04 | 0.04 | 0.00 | 0.01 | 0.00 | 0.00 | 0.00 | 0.00 |
| Proteobacteria_Gammaproteobacteria_Oceanospirillales_Oceanospirillaceae | 0.44 | 0.36 | 0.46 | 0.55 | 0.63 | 0.63 | 0.37 | 0.33 | 0.43 | 0.42 | 0.56 | 0.68 | 0.19 | 0.16 | 1.10 | 0.97 | 0.99 | 0.86 | 0.92 | 1.05 | 0.43 | 0.37 |
| Proteobacteria_Gammaproteobacteria_Oceanospirillales_Oleiphilaceae | 0.00 | 0.01 | 0.00 | 0.01 | 0.01 | 0.01 | 0.00 | 0.00 | 0.00 | 0.00 | 0.00 | 0.01 | 0.04 | 0.08 | 0.01 | 0.04 | 0.01 | 0.00 | 0.00 | 0.00 | 0.00 | 0.00 |
| Proteobacteria_Gammaproteobacteria_Oceanospirillales_OM182_clade | 0.06 | 0.05 | 0.05 | 0.04 | 0.12 | 0.13 | 0.88 | 0.75 | 0.57 | 0.49 | 0.18 | 0.19 | 1.01 | 0.76 | 1.93 | 1.87 | 0.50 | 0.33 | 0.19 | 0.32 | 1.03 | 0.85 |
| Proteobacteria_Gammaproteobacteria_Oceanospirillales_SAR86_clade | 21.88 | 19.29 | 17.58 | 21.06 | 9.35 | 9.48 | 9.35 | 8.31 | 8.58 | 8.20 | 13.37 | 14.95 | 16.20 | 19.47 | 10.92 | 12.80 | 14.67 | 15.02 | 13.75 | 12.13 | 15.15 | 15.31 |
| Proteobacteria_Gammaproteobacteria_Oceanospirillales_SS1-B-06-26 | 0.00 | 0.00 | 0.00 | 0.00 | 0.00 | 0.00 | 0.00 | 0.00 | 0.00 | 0.00 | 0.00 | 0.00 | 0.00 | 0.00 | 0.01 | 0.01 | 0.00 | 0.00 | 0.00 | 0.00 | 0.01 | 0.01 |
| Proteobacteria_Gammaproteobacteria_Pasteurellales_Pasteurellaceae | 0.00 | 0.00 | 0.00 | 0.00 | 0.00 | 0.00 | 0.00 | 0.00 | 0.00 | 0.00 | 0.00 | 0.00 | 0.00 | 0.00 | 0.00 | 0.00 | 0.00 | 0.00 | 0.00 | 0.00 | 0.00 | 0.00 |
| Proteobacteria_Gammaproteobacteria_Pseudomonadales_Moraxellaceae | 0.01 | 0.00 | 0.01 | 0.00 | 0.01 | 0.02 | 0.03 | 0.06 | 0.21 | 0.16 | 0.03 | 0.03 | 0.16 | 0.10 | 0.26 | 0.14 | 0.63 | 0.20 | 0.23 | 0.42 | 0.12 | 0.12 |
| Proteobacteria_Gammaproteobacteria_Pseudomonadales_Pseudomonadaceae | 0.01 | 0.01 | 0.02 | 0.00 | 0.01 | 0.01 | 0.02 | 0.00 | 0.04 | 0.08 | 0.00 | 0.00 | 0.01 | 0.00 | 0.00 | 0.01 | 0.03 | 0.01 | 0.00 | 0.03 | 0.00 | 0.01 |
| Proteobacteria_Gammaproteobacteria_Salinisphaerales_Salinisphaeraceae | 0.03 | 0.02 | 0.04 | 0.02 | 0.26 | 0.32 | 0.51 | 0.35 | 0.21 | 0.31 | 0.35 | 0.46 | 0.07 | 0.05 | 0.58 | 0.76 | 0.17 | 0.23 | 0.12 | 0.08 | 0.54 | 0.40 |
| Proteobacteria_Gammaproteobacteria_Sva0071_Sva0071 | 0.00 | 0.00 | 0.00 | 0.00 | 0.00 | 0.00 | 0.00 | 0.00 | 0.00 | 0.00 | 0.00 | 0.00 | 0.00 | 0.00 | 0.00 | 0.00 | 0.00 | 0.00 | 0.00 | 0.00 | 0.00 | 0.00 |
| Proteobacteria_Gammaproteobacteria_Thiotrichales | 0.00 | 0.01 | 0.00 | 0.00 | 0.00 | 0.00 | 0.00 | 0.00 | 0.00 | 0.00 | 0.00 | 0.01 | 0.00 | 0.00 | 0.00 | 0.00 | 0.00 | 0.00 | 0.00 | 0.00 | 0.00 | 0.00 |
| Proteobacteria_Gammaproteobacteria_Thiotrichales | 0.01 | 0.02 | 0.02 | 0.03 | 0.03 | 0.01 | 0.00 | 0.00 | 0.00 | 0.00 | 0.01 | 0.02 | 0.01 | 0.00 | 0.01 | 0.02 | 0.01 | 0.05 | 0.00 | 0.00 | 0.01 | 0.02 |
| Proteobacteria_Gammaproteobacteria_Thiotrichales_Francisellaceae | 0.00 | 0.00 | 0.01 | 0.01 | 0.04 | 0.05 | 0.00 | 0.00 | 0.00 | 0.00 | 0.01 | 0.04 | 0.01 | 0.01 | 0.00 | 0.00 | 0.00 | 0.00 | 0.00 | 0.00 | 0.00 | 0.01 |
| Proteobacteria_Gammaproteobacteria_Thiotrichales_Piscirickettsiaceae | 0.00 | 0.02 | 0.00 | 0.00 | 0.00 | 0.00 | 0.00 | 0.00 | 0.00 | 0.00 | 0.00 | 0.00 | 0.00 | 0.00 | 0.00 | 0.00 | 0.04 | 0.05 | 0.00 | 0.02 | 0.00 | 0.01 |
| Proteobacteria_Gammaproteobacteria_Thiotrichales_Thiotrichaceae | 0.29 | 0.17 | 0.36 | 0.18 | 0.32 | 0.39 | 0.15 | 0.07 | 0.08 | 0.13 | 0.31 | 0.31 | 0.03 | 0.07 | 0.02 | 0.04 | 0.13 | 0.19 | 0.10 | 0.14 | 0.03 | 0.03 |
| Proteobacteria_Gammaproteobacteria_Thiotrichales_Thiotrichales_Incertae_Sedis | 0.03 | 0.03 | 0.01 | 0.03 | 0.05 | 0.01 | 0.01 | 0.01 | 0.02 | 0.07 | 0.00 | 0.04 | 0.01 | 0.01 | 0.01 | 0.04 | 0.03 | 0.01 | 0.03 | 0.03 | 0.01 | 0.01 |
| Proteobacteria_Gammaproteobacteria_unknown | 0.00 | 0.00 | 0.00 | 0.00 | 0.00 | 0.00 | 0.00 | 0.00 | 0.00 | 0.00 | 0.00 | 0.00 | 0.00 | 0.00 | 0.00 | 0.00 | 0.00 | 0.00 | 0.00 | 0.00 | 0.00 | 0.00 |
| Proteobacteria_Gammaproteobacteria_unknown | 0.14 | 0.17 | 0.16 | 0.28 | 0.52 | 0.36 | 0.22 | 0.25 | 0.12 | 0.14 | 0.27 | 0.39 | 0.10 | 0.12 | 0.13 | 0.13 | 0.07 | 0.08 | 0.05 | 0.12 | 0.07 | 0.09 |
| Proteobacteria_Gammaproteobacteria_unknown | 1.23 | 1.22 | 1.06 | 1.25 | 1.82 | 1.35 | 0.93 | 0.82 | 0.38 | 0.69 | 0.83 | 1.48 | 0.36 | 0.67 | 0.04 | 0.14 | 0.70 | 1.33 | 1.72 | 1.06 | 0.14 | 0.21 |
| Proteobacteria_Gammaproteobacteria_Vibrionales_Vibrionaceae | 0.48 | 0.27 | 1.17 | 0.30 | 0.20 | 0.23 | 0.06 | 0.08 | 0.04 | 0.05 | 0.05 | 0.05 | 0.06 | 0.06 | 0.06 | 0.10 | 0.05 | 0.10 | 0.07 | 0.06 | 0.04 | 0.02 |
| Proteobacteria_Gammaproteobacteria_Xanthomonadales_JTB255_marine_benthic_group | 0.87 | 0.64 | 0.77 | 0.96 | 0.74 | 0.87 | 0.30 | 0.28 | 0.26 | 0.17 | 0.53 | 0.67 | 0.32 | 0.32 | 0.13 | 0.16 | 1.11 | 0.98 | 0.69 | 0.93 | 0.26 | 0.27 |
| Proteobacteria_Gammaproteobacteria_Xanthomonadales_Xanthomonadaceae | 0.00 | 0.00 | 0.00 | 0.01 | 0.01 | 0.00 | 0.01 | 0.02 | 0.06 | 0.07 | 0.00 | 0.00 | 0.01 | 0.03 | 0.10 | 0.06 | 0.08 | 0.05 | 0.44 | 0.26 | 0.11 | 0.07 |
| Proteobacteria_SC3-20 | 0.00 | 0.00 | 0.00 | 0.00 | 0.00 | 0.00 | 0.06 | 0.05 | 0.00 | 0.01 | 0.00 | 0.00 | 0.01 | 0.02 | 0.00 | 0.00 | 0.00 | 0.00 | 0.00 | 0.00 | 0.00 | 0.00 |
| Proteobacteria_SPOTSOCT00m83 | 0.03 | 0.06 | 0.08 | 0.04 | 0.13 | 0.13 | 0.26 | 0.26 | 0.03 | 0.02 | 0.09 | 0.17 | 0.02 | 0.06 | 0.01 | 0.00 | 0.14 | 0.10 | 0.12 | 0.08 | 0.02 | 0.02 |
| Proteobacteria_unknown | 0.00 | 0.00 | 0.00 | 0.00 | 0.01 | 0.02 | 0.02 | 0.00 | 0.00 | 0.00 | 0.01 | 0.01 | 0.00 | 0.00 | 0.00 | 0.00 | 0.00 | 0.01 | 0.02 | 0.02 | 0.00 | 0.00 |
| Proteobacteria_unknown | 0.03 | 0.02 | 0.04 | 0.02 | 0.01 | 0.00 | 0.00 | 0.00 | 0.00 | 0.00 | 0.01 | 0.01 | 0.00 | 0.00 | 0.00 | 0.00 | 0.00 | 0.02 | 0.00 | 0.00 | 0.00 | 0.00 |
| Proteobacteria_unknown | 0.17 | 0.19 | 0.39 | 0.41 | 0.19 | 0.15 | 0.22 | 0.28 | 0.11 | 0.09 | 0.13 | 0.15 | 0.18 | 0.20 | 0.10 | 0.05 | 0.11 | 0.08 | 0.35 | 0.29 | 0.03 | 0.09 |
| Saccharibacteria | 0.00 | 0.00 | 0.00 | 0.00 | 0.00 | 0.01 | 0.00 | 0.00 | 0.00 | 0.00 | 0.00 | 0.00 | 0.01 | 0.01 | 0.00 | 0.01 | 0.00 | 0.00 | 0.00 | 0.00 | 0.00 | 0.00 |
| Saccharibacteria_Unknown | 0.00 | 0.00 | 0.00 | 0.00 | 0.01 | 0.00 | 0.00 | 0.00 | 0.00 | 0.00 | 0.00 | 0.00 | 0.00 | 0.00 | 0.00 | 0.00 | 0.00 | 0.00 | 0.00 | 0.00 | 0.00 | 0.00 |
| Tenericutes_Mollicutes_Acholeplasmatales_Acholeplasmataceae | 0.00 | 0.01 | 0.00 | 0.00 | 0.00 | 0.00 | 0.00 | 0.00 | 0.00 | 0.00 | 0.00 | 0.00 | 0.00 | 0.00 | 0.00 | 0.00 | 0.00 | 0.00 | 0.00 | 0.00 | 0.00 | 0.00 |
| Tenericutes_Mollicutes_Entomoplasmatales_Incertae_Sedis | 0.00 | 0.00 | 0.00 | 0.00 | 0.00 | 0.00 | 0.00 | 0.00 | 0.00 | 0.00 | 0.00 | 0.00 | 0.00 | 0.00 | 0.00 | 0.00 | 0.00 | 0.00 | 0.00 | 0.00 | 0.00 | 0.00 |
| Tenericutes_Mollicutes_Mycoplasmatales_Mycoplasmataceae | 0.00 | 0.00 | 0.00 | 0.01 | 0.00 | 0.00 | 0.00 | 0.00 | 0.00 | 0.00 | 0.00 | 0.00 | 0.00 | 0.00 | 0.00 | 0.01 | 0.00 | 0.00 | 0.00 | 0.00 | 0.00 | 0.00 |
| TM6 | 0.00 | 0.01 | 0.01 | 0.01 | 0.00 | 0.00 | 0.00 | 0.00 | 0.00 | 0.00 | 0.00 | 0.00 | 0.00 | 0.00 | 0.00 | 0.00 | 0.00 | 0.00 | 0.00 | 0.00 | 0.00 | 0.00 |
| Verrucomicrobia_Arctic97B-4_marine_group | 0.04 | 0.07 | 0.14 | 0.11 | 0.12 | 0.06 | 0.13 | 0.12 | 0.01 | 0.01 | 0.12 | 0.13 | 0.00 | 0.01 | 0.06 | 0.04 | 0.09 | 0.07 | 0.12 | 0.09 | 0.02 | 0.02 |
| Verrucomicrobia_OPB35_soil_group | 0.04 | 0.02 | 0.03 | 0.06 | 0.07 | 0.08 | 0.38 | 0.39 | 0.02 | 0.03 | 0.25 | 0.23 | 0.06 | 0.04 | 0.14 | 0.10 | 0.18 | 0.12 | 0.10 | 0.12 | 0.02 | 0.02 |
| Verrucomicrobia_Opitutae | 0.00 | 0.00 | 0.00 | 0.00 | 0.01 | 0.00 | 0.00 | 0.00 | 0.00 | 0.00 | 0.00 | 0.00 | 0.00 | 0.00 | 0.01 | 0.02 | 0.00 | 0.00 | 0.00 | 0.00 | 0.01 | 0.01 |
| Verrucomicrobia_Opitutae_MB11C04_marine_group | 0.02 | 0.04 | 0.13 | 0.12 | 0.20 | 0.09 | 0.29 | 0.34 | 0.02 | 0.01 | 0.15 | 0.14 | 0.60 | 0.82 | 0.18 | 0.19 | 0.06 | 0.06 | 0.09 | 0.06 | 0.07 | 0.06 |
| Verrucomicrobia_Opitutae_Opitutales_Opitutaceae | 0.00 | 0.00 | 0.00 | 0.00 | 0.00 | 0.00 | 0.01 | 0.01 | 0.00 | 0.00 | 0.00 | 0.00 | 0.00 | 0.00 | 0.00 | 0.00 | 0.00 | 0.00 | 0.00 | 0.00 | 0.00 | 0.00 |
| Verrucomicrobia_Opitutae_Puniceicoccales_Puniceicoccaceae | 0.00 | 0.00 | 0.00 | 0.00 | 0.07 | 0.03 | 0.32 | 0.27 | 0.01 | 0.00 | 0.07 | 0.06 | 0.06 | 0.10 | 0.02 | 0.02 | 0.05 | 0.04 | 0.03 | 0.05 | 0.01 | 0.01 |
| Verrucomicrobia_Opitutae_RS-B22 | 0.00 | 0.00 | 0.01 | 0.02 | 0.00 | 0.01 | 0.00 | 0.00 | 0.00 | 0.00 | 0.00 | 0.00 | 0.00 | 0.00 | 0.00 | 0.00 | 0.00 | 0.00 | 0.00 | 0.00 | 0.00 | 0.00 |
| Verrucomicrobia_Verrucomicrobiae_Verrucomicrobiales_DEV007 | 0.07 | 0.08 | 0.14 | 0.12 | 0.02 | 0.04 | 0.02 | 0.03 | 0.01 | 0.00 | 0.05 | 0.04 | 0.00 | 0.00 | 0.01 | 0.01 | 0.03 | 0.03 | 0.02 | 0.08 | 0.01 | 0.00 |
| Verrucomicrobia_Verrucomicrobiae_Verrucomicrobiales_Rubritaleaceae | 0.01 | 0.02 | 0.01 | 0.00 | 0.00 | 0.01 | 0.01 | 0.01 | 0.00 | 0.00 | 0.01 | 0.00 | 0.01 | 0.02 | 0.00 | 0.00 | 0.00 | 0.01 | 0.00 | 0.00 | 0.01 | 0.04 |
| Verrucomicrobia_Verrucomicrobiae_Verrucomicrobiales_Verrucomicrobiaceae | 0.05 | 0.06 | 0.15 | 0.15 | 0.22 | 0.21 | 0.54 | 0.90 | 0.54 | 0.46 | 0.16 | 0.11 | 0.39 | 0.22 | 0.47 | 0.43 | 0.39 | 0.20 | 0.16 | 0.42 | 0.12 | 0.12 |
