# Supplementary material for: Bacterial Community Composition in the Sea Surface Microlayer Off the Peruvian Coast
Source: Front Microbiol. 2018 Nov 15;9:2699. doi: 10.3389/fmicb.2018.02699 (PMC6249803; doi:10.3389/fmicb.2018.02699)
Supplement: Supplementary file 5 [file Table_2.DOCX]

Supplementary Material

# Bacterial Community Composition in the Sea Surface Microlayer off the Peruvian Coast

**Birthe Zäncker^1*^, Michael Cunliffe^2,3^, Anja Engel^1^**

^1^GEOMAR – Helmholtz Centre for Ocean Research Kiel, Kiel, Germany

^2^Marine Biological Association of the UK, The Laboratory, Citadel Hill, Plymouth, UK

^3^Marine Biology and Ecology Research Centre, School of Biological and Marine Sciences, Plymouth University, Plymouth, UK

***Correspondence:**

Birthe Zäncker

bzaencker@posteo.de

# Supplementary Table

Table S2: Overview of diversity indices in the SML and the ULW across stations based on all OTUs detected in the samples.

| \| **Diversity index** \| **1** \| **2** \| **3** \| **4** \| **5** \| **6** \| **7** \| **8** \| **9** \| **10** \| **11** \| \| --- \| --- \| --- \| --- \| --- \| --- \| --- \| --- \| --- \| --- \| --- \| --- \| \| SML.shannon \| 2.62 \| 2.65 \| 2.71 \| 2.63 \| 2.55 \| 2.67 \| 2.56 \| 2.29 \| 2.69 \| 2.66 \| 2.25 \| \| SML.simpson \| 0.89 \| 0.91 \| 0.91 \| 0.91 \| 0.89 \| 0.91 \| 0.90 \| 0.86 \| 0.91 \| 0.91 \| 0.85 \| \| SML.invsimpson \| 9.36 \| 10.63 \| 11.75 \| 11.11 \| 9.11 \| 11.55 \| 9.70 \| 7.37 \| 11.58 \| 11.65 \| 6.79 \| \| ULW.shannon \| 2.64 \| 2.61 \| 2.74 \| 2.64 \| 2.64 \| 2.69 \| 2.56 \| 2.36 \| 2.67 \| 2.68 \| 2.35 \| \| ULW.simpson \| 0.90 \| 0.90 \| 0.92 \| 0.91 \| 0.90 \| 0.92 \| 0.90 \| 0.87 \| 0.91 \| 0.92 \| 0.87 \| \| ULW.invsimpson \| 10.17 \| 9.76 \| 12.50 \| 10.75 \| 10.49 \| 11.86 \| 9.69 \| 7.99 \| 11.43 \| 12.02 \| 7.58 \| |
| --- | --- | --- | --- | --- | --- | --- | --- | --- | --- | --- | --- | --- | --- | --- | --- | --- | --- | --- | --- | --- | --- | --- | --- | --- | --- | --- | --- | --- | --- | --- | --- | --- | --- | --- | --- | --- | --- | --- | --- | --- | --- | --- | --- | --- | --- | --- | --- | --- | --- | --- | --- | --- | --- | --- | --- | --- | --- | --- | --- | --- | --- | --- | --- | --- | --- | --- | --- | --- | --- | --- | --- | --- | --- | --- | --- | --- | --- | --- | --- | --- | --- | --- | --- | --- |
|  |
|  |
|  |
|  |
|  |
|  |
|  |
|  |
|  |
|  |
|  |
