# Supplementary material for: Bacterial Community Composition in the Sea Surface Microlayer Off the Peruvian Coast
Source: Front Microbiol. 2018 Nov 15;9:2699. doi: 10.3389/fmicb.2018.02699 (PMC6249803; doi:10.3389/fmicb.2018.02699)
Supplement: Supplementary file 6 [file Table_3.DOCX]

Supplementary Material

# Bacterial Community Composition in the Sea Surface Microlayer off the Peruvian Coast

**Birthe Zäncker^1*^, Michael Cunliffe^2,3^, Anja Engel^1^**

^1^GEOMAR – Helmholtz Centre for Ocean Research Kiel, Kiel, Germany

^2^Marine Biological Association of the UK, The Laboratory, Citadel Hill, Plymouth, UK

^3^Marine Biology and Ecology Research Centre, School of Biological and Marine Sciences, Plymouth University, Plymouth, UK

***Correspondence:**

Birthe Zäncker

bzaencker@posteo.de

# Supplementary Table

**Table S3:** Concentrations of total and dissolved carbohydrates (T/DCHO) and bacterial abundances across stations during SO243.

| **Station** | **TCHO_SML_**  **[nmol l^-1^]** | **TCHO_ULW_**  **[nmol l^-1^]** | **DCHO_SML_**  **[nmol l^-1^]** | **DCHO_ULW_**  **[nmol l^-1^]** | **Bacteria_SML_**  **[10^5^ cells ml^-1^]** | **Bacteria_ULW_**  **[10^5^ cells ml^-1^]** |
| --- | --- | --- | --- | --- | --- | --- |
| 1 | 908.9 | 532.6 | 552.9 | 469.8 | 5.7 | 4.4 |
| 2 | 827.7 | 560.8 | 656.1 | 550.8 | 7.9 | 6.9 |
| 3 | 543.3 | 575.7 | 471.8 | 461.4 | 4.6 | 5.8 |
| 4 | 564.7 | 528.9 | 467.2 | 467.1 | 17.5 | 18.9 |
| 5 | 1204.5 | 1495.0 | 696.3 | 1200.0 | 9.7 | 10.0 |
| 6 | 561.1 | 570.9 | 454.4 | 417.0 | 10.1 | 10.0 |
| 7 | 738.0 | 763.6 | 518.3 | 495.9 | 17.2 | 18.0 |
| 8 | 765.7 | 1389.4 | 351.8 | 529.0 | 6.6 | 6.9 |
| 9 | 654.8 | 672.6 | 385.3 | 500.6 | 5.1 | 6.1 |
| 10 | 550.0 | 463.0 | 416.8 | 455.9 | 7.5 | 7.5 |
| 11 | 592.3 | 767.7 | 384.8 | 288.3 | 5.9 | 6.4 |
